# Glioblastoma microenvironment and its reprogramming by oncolytic virotherapy

**DOI:** 10.3389/fncel.2022.819363

**Published:** 2022-09-09

**Authors:** Zhongbing Qi, Xiangyu Long, Jiyan Liu, Ping Cheng

**Affiliations:** ^1^Department of State Key Laboratory of Biotherapy and Cancer Center/Collaborative Innovation Center for Biotherapy, West China Hospital, Sichuan University, Chengdu, China; ^2^Department of Biotherapy, Cancer Center, West China Hospital of Sichuan University, Chengdu, China; ^3^Department of Oncology, West China Guang’an Hospital, Sichuan University, Guangan, China

**Keywords:** glioblastoma, oncolytic virus, tumor microenvironment, anti-tumor immunity, combination therapy

## Abstract

Glioblastoma (GBM), a highly aggressive form of brain tumor, responds poorly to current conventional therapies, including surgery, radiation therapy, and systemic chemotherapy. The reason is that the delicate location of the primary tumor and the existence of the blood-brain barrier limit the effectiveness of traditional local and systemic therapies. The immunosuppressive status and multiple carcinogenic pathways in the complex GBM microenvironment also pose challenges for immunotherapy and single-targeted therapy. With an improving understanding of the GBM microenvironment, it has become possible to consider the immunosuppressive and highly angiogenic GBM microenvironment as an excellent opportunity to improve the existing therapeutic efficacy. Oncolytic virus therapy can exert antitumor effects on various components of the GBM microenvironment. In this review, we have focused on the current status of oncolytic virus therapy for GBM and the related literature on antitumor mechanisms. Moreover, the limitations of oncolytic virus therapy as a monotherapy and future directions that may enhance the field have also been discussed.

## Introduction

Glioblastoma (GBM) is the most common and aggressive form of primary brain tumor. For patients with newly diagnosed GBM, the most current standard of care is surgical resection of the main part of the tumor, followed by radiotherapy (RT) and adjuvant temozolomide (TMZ; Stupp et al., 2005). Despite these multiple approaches, the median survival for patients with GBM is still less than 24 months (Delgado-López and Corrales-García, 2016). The recurrence rate of GBM is certainly 100% (Davis, 2016) and survival after relapse is less than 12 months. So far, several regimens explored in a clinic setting, including immunotherapy and targeted therapy, have shown no significant survival advantage with either single or combination therapy relative to that with the conventional therapy (Haines and Gabor Miklos, 2014; Diaz et al., 2017), which can majorly be attributed to the unique microenvironment of GBM (Brown et al., 2016; O’Rourke et al., 2017; Omuro et al., 2018). Hence, novel therapeutic options for treating GBM are imperatively needed.

Oncolytic virus (OV) therapy has attracted great attention as a novel treatment modality for GBM. OVs are antitumor agents that have been designed or selected to replicate in and selectively kill tumor cells. In the early development of OVs, researchers mainly focused on their selective ability and the direct lysis of tumor cells. The evolving understanding of the tumor immune microenvironment has shifted the attention of researchers to the stimulating effect of OVs on the immune system (Lichty et al., 2014). The virus itself and the release of damage-related molecular patterns (DAMPs) and tumor-associated antigens (TAAs) in the process of virus-induced tumor cell lysis correspondingly led to the activation of innate and adaptive immune responses, thereby inducing an antitumor immune response. This immunostimulatory ability of OVs in the GBM microenvironment is likely to turn the “cold” immune microenvironment into a “hot” one (Martikainen and Essand, 2019). Moreover, as excellent gene-delivery vehicles, OVs can introduce specific therapeutic genes (including tumor-suppressor genes, immunostimulatory genes, and anti-angiogenic genes) into tumor cells to execute their expression (Gatson et al., 2012; Loskog, 2015).

This review summarizes the need for establishing the GBM microenvironment for improving the efficacy of the current oncolytic virotherapy. We have focused on the effects of OVs on the GBM microenvironment in combination with the anti-tumor mechanism of OVs, which includes tumor parenchyma cells, stromal cells, and immune cells. Meanwhile, we have discussed the possible future development directions in this field in response to the challenges of oncolytic virotherapy as a monotherapy.

## GBM Microenvironment and The Current Needs for OVs

GBM has a high degree of inter- and intratumor heterogeneities. Interactions between the different components of the GBM microenvironment further enhance this diversity, while making it the most challenging and meaningful therapeutic target (Quail and Joyce, 2017). The GBM microenvironment consists of GBM cells, glioblastoma stem cells (GSCs), CNS resident cells, fibroblasts, endothelial cells, pericytes, and immune cells. Among these, endothelial cells, pericytes, and astrocyte foot processes constitute the blood–brain barrier (BBB; Abbott, 2013). In addition, the unique extracellular matrix (ECM) formed by the interaction of CNS resident cells with macromolecules such as proteins and polysaccharides are also intricately linked to the tumor microenvironment.

The tumor microenvironment is the main reason for the unsatisfactory therapeutic effect of GBM. Therefore, resolving these issues is critical to the application of GBM in oncolytic virotherapy. First, the fact that the BBB only allows the passage of small lipophilic molecules greatly limits the delivery of systemic therapeutic drugs. Although tumor formation disrupts the integrity of the BBB, which results in drug leakage and achieves some clinical efficacy, most aggressive tumor cells are protected by the intact endothelial cells layer and can freely cross the BBB (Eichler et al., 2011). In addition, the presence of the BBB contributes to the immune privilege of the central nervous system, which again greatly limits the ability of T cells to exert their antitumor effects (Jackson et al., 2014). Second, GSCs, as tumor cells with self-renewal and multi-directional differentiation characteristics, confer enhanced chemo- and radio-resistance to GBM (Ahmad and Amiji, 2017). More importantly, the immune cells within the GBM microenvironment act as both a hindrance and an opportunity in the GBM treatment. As the main infiltrating immune cell population, tumor-associated macrophages (TAMs) function to promote tumor growth and induce T-cell dysfunction and immunosuppression together with myeloid-derived suppressor cells (MDSCs) and CD4^+^CD25^+^Foxp3^+^T-regulatory cells (Tregs; Gabrilovich and Nagaraj, 2009). However, T-cells that infiltrate GBM, despite the small number, are among the immune cells with the greatest potential for antitumor effects. The clinical outcomes suggest that T-cell infiltration has an important positive effect on the survival of GBM patients (Lohr et al., 2011). M1 macrophages exert an adaptive antitumor immune response by presenting antigens to the T cells.

## Characteristics of OV Vectors for GBM Treatment

**Figure 1:**
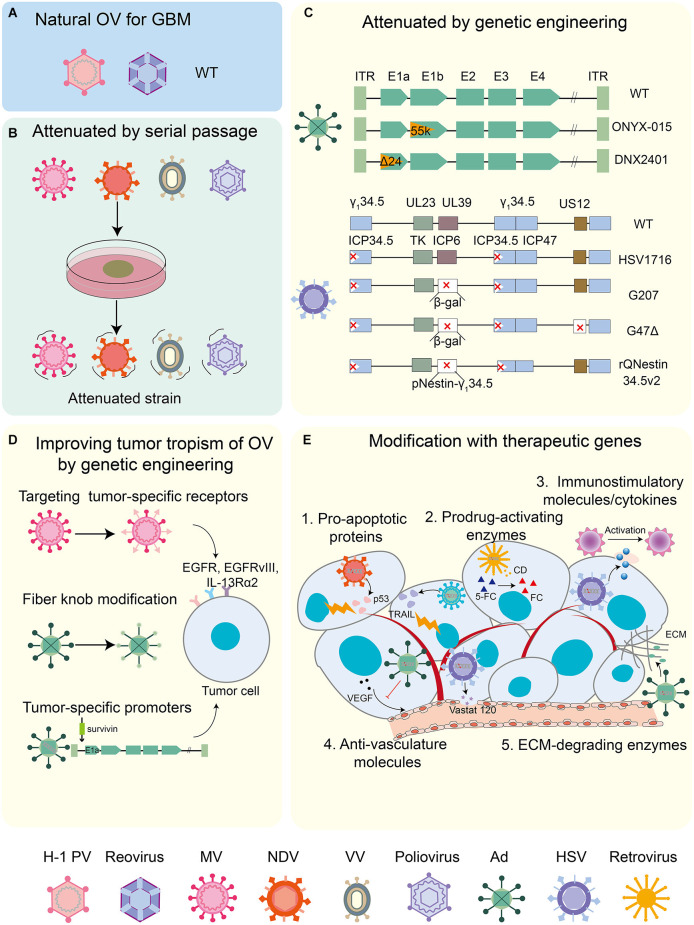
OVs in GBM treatment. **(A)** Natural OVs for GBM. Wild-type (WT) virus strains including H1 PV and reovirus could be used directly for oncolytic virotherapy of GBM. **(B)** OVs attenuated by serial passaging include MV, NDV, VV, and poliovirus. **(C)** OVs attenuated by genetic engineering. Improving the safety of Ad and HSV by deleting virulence genes. **(D)** Enhanced tropism of OV *via* genetic engineering. MV-targeted entry into tumors is facilitated by targeting tumor-associated specific receptors (such as EGFR, EGFRvIII, and IL-13Rα2 receptor). Enhanced tumor targeting of Ad by modifying fiber knobs of viral capsid proteins (exchanging viral capsid protein fibers of different serotypes and RGD modifications). Ad replicating in tumor cells, but not in normal cells, is controlled by tumor-specific promoter sequences (such as surviving) into the genetic sequence of a virus. **(E)** Enhanced antitumor effects of OV by modifying with different therapeutic genes, such as pro-apoptotic proteins, conditional cytotoxic enzymes, immunomodulatory factors/cytokines, anti-angiogenic molecules, and hyaluronidase.

OVs can be classified into two types: (1) wild-type virus strains or naturally attenuated strains, which are employed for OV research without any modification, or strains that have been subjected to continuous passage in the natural state, such as Parvovirus H1-ParvOryx01 (H-1PV), reovirus, measles virus (MV), Newcastle disease virus (NDV), and vaccinia virus (VV; Lan et al., 2020), (2) genetically engineered OVs that have been genetically modified by deleting the virulence genes or by inserting foreign genes. There are three purposes of genetic modification of OV: to enhance the safety of OV, to improve the tropism of OV to tumor cells, and to enhance the anti-tumor effect of OV (Figure 1). Here, we have summarized several commonly used OVs for the GBM treatment (Table 1).

**Table 1:** Some details on oncolytic viruses used in GBM therapy.

**Virus**	**Deriving from**	**Baltimore classification**	**Virion**	**Capsid symmetry**	**Cell receptor**	**Neurovirulence factor**	**Penetrating the blood-brain barrier**	**Bystander effect**	**Targeting GSCs**
Herpesvirus 1	Human pathogen	Group I: dsDNA	Enveloped	Icosahedral	HVEM, nectin 1, and nectin 2	ICP34.5	-	+	+
Adenovirus		Group I: dsDNA	Naked	Icosahedral	CAR	Not applicable	-	+	-
Reovirus		Group III: dsRNA	Naked	Icosahedral	unknown	Not applicable	+	+	+
Zika virus		Group IV: ss (+) RNA	Enveloped	Icosahedral	AXL	unknown	+	+	+
Vaccinia virus	Human vaccine strains	Group 1: dsDNA	Complex coat	Complex	unknown	Not applicable	-	unknown	+
Measles virus		Group V: ss (-) RNA	Enveloped	Icosahedral	SLAM and CD46	unknown	-	+	-
Poliovirus		Group IV: ss (+) RNA	Naked	Icosahedral	CD155	unknown	+	+	-
Vesicular stomatitis virus	Non-human pathogen	Group V: ss (-) RNA	Enveloped	Helical	LDLR	unknown	-	unknown	-
Newcastle disease virus		Group V: ss (-) RNA	Enveloped	Helical	unknown	unknown	+	+	+
Myxoma virus		Group I: dsDNA	Enveloped	Compound	unknown	Not applicable	-	+	-
Parvovirus H1		Group II: ssDNA	Naked	Icosahedral	Sialic acid residues	Not applicable	+	+	-

### Oncolytic H-1 PV

Oncolytic H-1PV is a rodent-derived non-enveloped single-stranded DNA virus and the smallest OV. Compared with other OVs, H-1PV has its advantages: (1) H-1PV can cross the blood-brain/tumor barrier from blood to tumor and (2) Humans lack pre-existing H-1PV-specific antibodies (Geletneky et al., 2017).Wild-type H-1PV infection results in the efficient killing of the human GBM cells (Herrero et al., 2004; Figure 1A). In addition, oncolytic H-1PV infection demonstrated the lysis of adult or pediatric GBM stem cell culture models (Josupeit et al., 2016). Past studies revealed that H-1PV triggers glioma death through the accumulation of lysosomal cathepsins B and L in the cytosol of infected cells and the downregulation of cystatin, a physiological inhibitor of cathepsins (Di Piazza et al., 2007). In addition, the transformative effect of H-1PV on the immunogenic tumor microenvironment makes it promising as immunotherapy for GBM (Angelova et al., 2017).

### Reovirus

Reovirus is an unenveloped, double-stranded RNA virus that is tropic to the GBM cells. Reoviruses enter the tumor cells primarily through the ligated adhesion molecule A (JAM-A) receptor (Gong and Mita, 2014). In the current *in vitro* and *in vivo* studies, wild-type reovirus revealed specificity and oncolytic activity against glioma cells (Wilcox et al., 2001; Figure 1A). In addition, reovirus has been reported to upregulate the PD-L1 expression, implying the potential of reovirus in combination with ICI for GBM (Samson et al., 2018).

### Oncolytic MV

MV is a non-enveloped single-stranded RNA virus. MVs enter the tumor cells through the overexpressed CD46 receptor on the surface of GBM cells without damaging the normal brain tissues (Yanagi, 2001). MV expressing carcinoembryonic antigen gene (MV-CEA) is an attenuated strain of MV used for human vaccination (Lin and Richardson, 2016). Subsequent analyses retargeted MVs to the epidermal growth factor receptor, EGF receptor variant III, and the IL-13Rα2 receptor to increase their specificity for GBM (Estevez-Ordonez et al., 2021; Figure 1C). The expression of the sodium iodide transporter MV (MV-NIS) not only tracks the viral infection efficiency but also potentially improves the therapeutic efficacy by allowing the intracellular uptake of I-131 (Aref et al., 2016).

### NDV

NDV is a single-stranded negative-sense RNA virus with selective oncolytic properties (Sinkovics and Horvath, 2000). NDV achieves oncolysis by activating the Ras pathway and the antitumor immune responses by increasing the TNF-α secretion level from the host immune cells. In addition, a preclinical analysis of recombinant NDV (rNDV-p53) delivering p53 oncolytic agents demonstrated that the recombinant virus inhibited GBM cell growth and invasiveness both *in vitro* and *in vivo* (Fan et al., 2018; Figure 1D).

### Oncolytic VV

VV is a large, double-stranded enveloped DNA virus (Wollmann et al., 2012). Strongly attenuated strains demonstrate impaired replication and are primarily used as gene therapy vectors. Less attenuated VV strains such as Western Reserve, Lister, and Copenhagen have been used as recombinantly engineered OVs (Figure 1A). The first VV for GBM-enhanced tumor selectivity by equipping *p53* (Gridley et al., 1998). In a recent study, a double-group VV, in which the *tk* and growth factor (*vgf*) genes were deleted and the *GM-CSF* and *lactaptin* genes were inserted demonstrated better therapeutic effects in human GBM (Vasileva et al., 2021).

### Poliovirus

Poliovirus is a positive-strand RNA virus, which is the causative agent of human polio. The neurotoxicity of poliovirus can be attributed to two factors: (1) selective binding to poliovirus receptors (Necl-5 or CD155) expressed on motor neurons and (2) internal ribosomal entry at the 5’ end of viral RNA locus (IRES) genome (Wollmann et al., 2012). Poliovirus has a tropism for GBM that highly expresses the CD155 receptor (Merrill et al., 2004). Most of the poliovirus used for oncolytic virotherapy uses its Sabin attenuated strain (Figure 1A). Several studies have demonstrated the therapeutic potential of attenuated oncolytic polio/rhinovirus recombinant (PVSPIRO) in patients with GBM (Banerjee et al., 2021).

### Oncolytic adenovirus

Adenovirus is a non-enveloped double-stranded DNA virus. Conditionally replicating adenoviruses (CRAds) involving gene deletion of E1A and E1B are commonly applied for OV studies, and the human adenovirus serotype 5 is currently the most widely used adenovirus vector (Figure 1B). *E1B*-deleted adenovirus ONYX-015 is the first CRAd to be tested in a phase-I clinical trial for recurrent glioma; it selectively replicates and lyses in tumor cells with an abnormal *p53* without damaging the normal cells (Shinoura et al., 1999; Chiocca et al., 2004). Another CRAd is DNX2401, which has a deletion of 24 bp in the E1A region; it replicates in tumor cells with mutations in *RB*. In addition, in order to overcome the difficulties of non-targeted replication of the virus and the lack of coxsackie-adenovirus receptor (CAR) on the surface of GBM cells, arginine-glycine-aspartic acid (RGD) modification was performed on the fiber portion of adenovirus-Δ24 to increase the ability of the virus to target the GBM cells (Fueyo et al., 2003). CRAd-S-pk7, also a CRAd for GBM, drives the expression of *E1A* critical for viral replication through the tumor-specific promoter survivin, and the adenovirus fibers were modified by incorporating the polyline sequences (Ulasov et al., 2007; Figure 1C).

### Oncolytic herpes simplex virus (oHSV)

oHSV, as an enveloped DNA virus, offers the advantage of inconsistent neurovirulence genes and oncolytic genes. Therefore, oHSV is the OV with the most variants for the treatment of GBM, including G207, G47Δ, HSV1716, and rQNestin-34.5 (Figure 1B). These mutants were designed to delete two copies of the neurovirulence gene γ134.5 or to disrupt *UL39* (Mineta et al., 1995). *UL39* encodes the ribonucleotide reductase (ICP 6) responsible for DNA replication in neurons. HSV1716, a first-generation mutant, contains an intact *UL39* but lacks two copies of *γ134.5* that mediates the PKR/eIF-2a signaling pathway and IFN-induced antiviral mechanisms. The second-generation oHSV—G207—is a mutant in which the two copies of *γ134.5* and *UL39* have been deleted. However, double deletion of the two copies of *γ134.5* resulted in the replication ability of G207 in the target cells. Thus, rQNestin 34.5v2, also a second-generation oHSV, deletes one copy under the control of the nestin promoter simultaneously as the double deletion of the *γ134.5* (Nakashima et al., 2015). Nestin is a hallmark molecule in gliomas and is not expressed in other neuronal cells (Zhao et al., 2022). Another mutant that overcomes the insufficient replication capacity of G207 is G47Δ, a third-generation oHSV. On the basis of G207, this mutant also deleted *α47* and the US11 promoter. When compared with G207, G47Δ has been reported to showcase enhanced replication ability and antitumor effect, and it can kill human-derived GSCs (Fukuhara et al., 2016). Presently, G47Δ (Delytact/Teserpaturev) has been approved in Japan for the treatment of malignant glioma patients, making it the world’s first OV product for brain tumors. In addition, G47Δ can also load the cytokine IL12, such as G47Δ-mIL12, which offers a distinct survival advantage in preclinical mouse glioblastoma studies (Cheema et al., 2013).

### Oncolytic retrovirus

Retroviruses are RNA viruses that contain two identical single-stranded positive strands; they were originally used as vectors for gene therapy (Garcia-Montojo et al., 2018). Subsequent analyses have demonstrated that the replication ability of retroviruses is critical for the delivery of their therapeutic genes (Logg et al., 2012). Vocimageneamiretrorepvec (Toca 511) is a Moloney murine leukemia virus (MLV) encoding *cytosine deaminase* (*CD*) that locally converts the prodrug 5-fluorocytosine (5-FC) to active 5-fluorouracil (5-FU) to cause the direct killing of GBM cells (Perez et al., 2012; Figure 1D).

#### Oncolytic zika virus (ZIKV)

ZIKV is a single-stranded positive-stranded RNA virus of the Flaviviridae family of Flaviviridae (Hancock et al., 2014). ZIKV is neurotropic, albeit the specific mechanism of brain invasion remains unclear. However, recent studies have demonstrated that ZIKV can cross the endothelial barrier *via* an endocytosis/exocytosis-dependent replication pathway or transcytosis without disrupting the BBB (Papa et al., 2017). Furthermore, ZIKV demonstrated an oncolytic effect on GSCs cells. The genetic engineering of ZIKV mainly targets *NS5*, which hinders the induction and transduction of IFN-I signaling (Su and Balasubramaniam, 2019). ZIKV with a mutation attenuates its replication in neuronal cells responsive to type-I IFN while maintaining the activity on GSCs (Zhu et al., 2017).

## Oncolytic Virotherapy and Other Virotherapies of GBM

**Figure 2:**
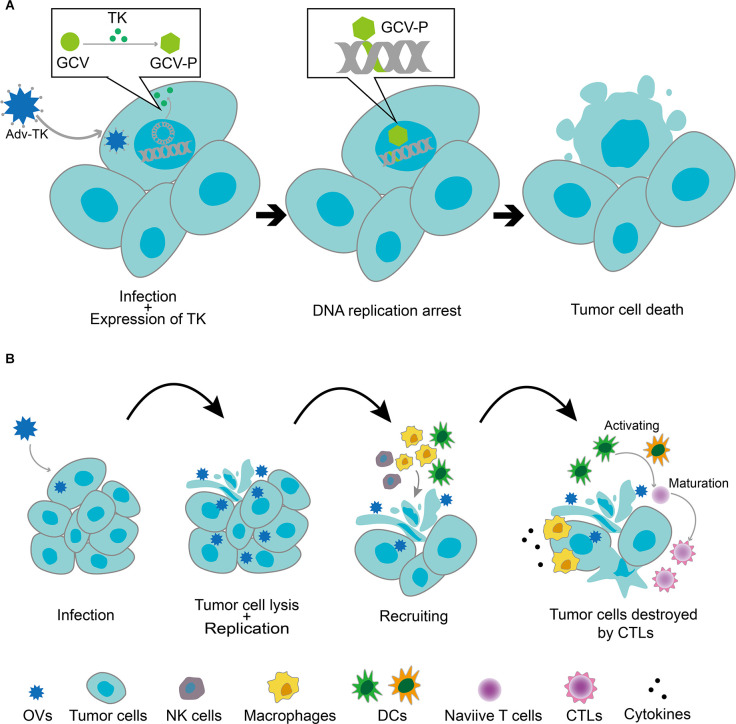
**(A)** Replication-deficient viral vectors infecting tumor cells and delivering anticancer genes. After the tumor cells were infected with a recombinant replication-deficient adenoviral vector expressing the thymidine kinase gene (*Adv-TK*), the *TK* gene was transcribed and translated into the nucleus. TK phosphorylated monophosphate nucleosides (e.g., ganciclovir and GCV) to triphosphate nucleosides, which then bonded to nascent DNA strands in the tumor cells, resulting in DNA replication arrest and tumor cell death. **(B)** Replicative viral vector (OV)-mediated antitumor mechanism. OVs selectively replicated in tumor cells and induced their lysis, releasing viral particles that further infected other tumor cells. Tumor cell lysis by OVs infection resulted in the release of tumor-associated antigens (TAAs), cell-derived damage-associated molecular patterns (DAMPs), and viral pathogen-associated molecular patterns (PAMPs), which recruited antigen-presenting cells (APCs) and innate immune cells (including NK cells and macrophages). Innate immune cells, such as macrophages, were activated and released cytokines, which led to the inflammation of the tumor microenvironment. APCs were activated by TAAs, DAMPs, PAMPs, pro-inflammatory cytokines, and chemokines and elicited an immune response mediated by anti-tumor cytotoxic CD8^+^ T lymphocytes (CTLs).

The main means of viral use for GBM therapy are viral vectors and oncolytic virotherapy. The viral vectors employed in genetic engineering therapy include non-replicating viral vectors and replicating viral vectors (OV; Figure 2). The main difference between the two is the ability of the virus to replicate. Non-replicating viral vectors use replication-incompetent viruses, which are used only to deliver therapeutically useful vectors by enhancing cellular function and targeting the abnormal cells. Commonly used non-replicating viral vectors for GBM therapy include non-replicating adenovirus, adeno-associated virus (AAV), retrovirus, and lentivirus (Banerjee et al., 2021). In addition to the field of tumor therapy, gene therapy non-replicating viral vectors also play an important role in other major human disease fields. In contrast, OVs use replication-competent viruses that are usually weakly pathogenic, such as non-human host viruses or pre-attenuated and modified human host viruses. These viruses tend to exhibit better targeting and oncolytic effects, leading to the direct lysis of tumor cells and the release of new viral particles through selective autonomous replication in tumor cells. Of course, the genetically modified OVs retain their ability to kill tumors and can be used as gene expression vectors for therapeutic genes. When compared with non-replicating viral vectors, OVs vectors that retain the ability to replicate can achieve better biodistribution in the tumor tissues through successive cycles of infection and replication (Boviatsis et al., 1994). In addition, tumor cell lysis by OVs infection results in the release of tumor-associated antigens (TAAs), cell-derived damage-associated molecular patterns (DAMPs), and viral pathogen-associated molecular patterns (PAMPs), which altogether recruit antigen-presenting cells (APCs) and innate immune cells (including NK cells and macrophages). Innate immune cells, such as macrophages, are activated and release cytokines that inflame the tumor microenvironment. APCs are activated by TAAs, DAMPs, PAMPs, pro-inflammatory cytokines, and chemokines and elicit an immune response mediated by anti-tumor cytotoxic CD8^+^ T lymphocytes (CTLs; Chiocca et al., 2019). The immunostimulatory effects of OVs make them excellent immune adjuvants to enhance immunotherapy for GBM (Lim et al., 2018). Non-replicating viral vectors, on the other hand, cannot induce strong immunity in the tumor microenvironment, and they can only generate anti-tumor immunity by adding additional genes.

Overall, this review focuses on the contribution of replication-competent viruses to the field of GBM.

## OVs Targeting Glioblastoma Parenchymal Cells

### GBM tropism and direct oncolysis of OVs

Consistent with the tumor-selective mechanism of general OVs, the selective entry of OVs into GBM cells is often mediated by multiple factors. First, the tumor cells often specifically express viral receptors for OVs. This is also the main reason why some early OVs have gained public attention. For example, MV and polio virus enter tumor cells by binding to CD46 and CD155, which are highly expressed on tumor cells (Anderson et al., 2004; Merrill et al., 2004). Second, the active metabolism in tumor cells greatly facilitates the replication of OVs. Third, the abnormal innate defense signaling pathways in tumor cells provide convenient conditions for virus replication. For example, reovirus utilizes its unique double-stranded RNA genome to be selective for tumors with the Ras pathway upregulation by interacting with the protein kinase R pathway (Nishikawa et al., 1994).

OV can be genetically modified by a variety of strategies to effectively enhance its tropism. Taking advantage of the inactivation of tumor-suppressor genes in tumor cells, the ability to target infection of tumor cells can be enhanced by mutating or knocking out certain genes to make them lose the ability to replicate in normal cells. Oncolytic adenovirus (oAd) oncorine, which lacks *E1B*-55 kDa, does not generally replicate in normal cells, rather it selectively replicates in *p53*-deficient tumors (Ady et al., 2014). Furthermore, OVs selectivity can be enhanced by loading tumor-specific promoters to deliver genes that are essential for OVs proliferation. Tissue-specific promoters driving the *E1A* expression to enhance oAd targeting include human telomerase reverse transcriptase promoter (hTERT), hypoxia response promoter (HRE), prostate-specific antigen promoter (PSA), alpha-feto protein promoter (AFP), alpha-lactalbumin promoter (ALA), and mucin 1 promoter (DF3/MUC1; Hardcastle et al., 2007; Tian et al., 2022). The modification of viral capsid proteins for tropism is also one of the means to enhance the accuracy of tumor targeting. The exchange of viral capsid protein fibers or the generation of fiber chimeras by swapping knob domains and axons are effective in modifying the tropism ofadenovirus (Jiang et al., 2006). Moreover, OVs can be engineered to retarget tumor-specific receptors for entry into the tumor cells. EGFR and HER2, which are highly expressed in human glioblastoma and other tumor cells, have been widely applied for OV engineering (Allen et al., 2006; Gambini et al., 2012).

After selectively entering tumor cells, OVs can directly lyse tumor cells and release new virus particles, thereby infecting new tumor cells without damaging the normal cells. There is currently no exact mechanism for direct cleavage. However, relevant recent literature suggests that exosomal vesicles after OVs that infect tumor cells also show tumor tropism (Kakiuchi et al., 2021).

GSCs are not only an important cause of GBM resistance to conventional therapies but also play an important role in the formation of tumor vasculature and are inseparable from the immunosuppressive GBM microenvironment, which makes them an important therapeutic target. The genetically attenuated and modified ZIKV strain selectively targets GSCs and exhibits good oncolytic activity (Zhu et al., 2017; Chen et al., 2018). Although the application of wild-type ZIKV to GBM is challenging, the existing studies have demonstrated the safety of the attenuated strain for normal tissues. In addition, another important study demonstrated that ZIKV distinguishes normal cells from GSCs by recognizing the SOX2-related downregulation of the innate antiviral immune response in GSCs (Zhu et al., 2020). Regarding other classic OVs, adenovirus has demonstrated its therapeutic potential against GSCs by inducing autophagy in GSCs (Jiang et al., 2007). The deletion of the key neurotoxic gene *γ34.5* restricts the HSV republication in GSCs (Peters et al., 2018). MG18L (HSV-1 with U_S_3 deletion and U_L_39 inactivation) can effectively replicate in GSCs and exhibit antitumor activity *in vivo* with severely reduced neurotoxicity (Kanai et al., 2011).

### Engineered OVs that enhance the ability to directly kill tumor cells

#### Expression of tumor-suppressor genes

Tumor-suppressor genes execute the functions of regulating the cell cycle, cell proliferation and death, and DNA damage repair system in tumor cells. Engineering OVs to restore inactive tumor suppressor genes in tumor cells within GBM is a common approach for OVs potentiation. Inactivation of the p53 gene is one of the most frequently mutated tumor suppressor genes in GBM. Preclinical studies have shown that the use of recombinant NDV to deliver P53-encoded tumor suppressor proteins effectively inhibited the growth and invasion of GBM cells *in vitro* and *in vivo* (Fan et al., 2018). In addition, phosphatase and tensin homolog (PTEN) is also a good tumor suppressor gene, and its inactivation is associated with aberrant activation of the PI3K pathway in tumors. Studies have shown that the resistance of PTEN-deficient GBM to ICB has become a major challenge for the clinical treatment of ICB therapy (Peng et al., 2016). However, the combination of OVs with the PI3K-AKT pathway inhibition can overcome this deficiency and effectively enhance the therapeutic efficacy of PD-1 blockers and PI3K inhibitors in PTEN-deficient tumors (Xing et al., 2021).

#### Expression of TRAIL

The induction of tumor cell apoptosis forms the basic strategy of anti-tumor drugs. Tumor necrosis factor-related apoptosis-inducing ligand (TRAIL) specifically induces apoptosis in a death receptor-dependent manner, thereby becoming a promising antitumor drug. The results from a preclinical analysis revealed that recombinant oncolytic HSV (oHSV-TRAIL) can efficiently kill chemoresistance-mediated GSCs and exhibited resistance in mouse GBM models derived from chemoresistant primary and recurrent GSCs’ tumor efficacy (Jahan et al., 2017). However, as a result of antiviral immunity, the clinical response rate of oHSV in phase I and Ib clinical trials of relapsed GBM is not ideal (Harrow et al., 2004; Markert et al., 2009). To circumvent this problem, another study used mesenchymal stem cells to deliver oHSV-TRAIL and examined the kinetics in a clinically applicable mouse model, which signified the clinical translatability of this therapy (Duebgen et al., 2014).

#### Expression of conditional cytotoxic genes

The carrier properties of OVs were designed to deliver conditional cytotoxic genes to arm OV in order to enhance its tumor-killing effect. This strategy is based on the selective expression of conditional cytotoxic enzymes in GBM cells and the conversion of nontoxic prodrugs into cytotoxic drugs. Numerous enzyme/prodrug combinations have been discovered and characterized for the treatment of GBM, which include herpes simplex virus-thymidine kinase (HSV-TK)/ganciclovir (GCV), cytosine deaminase (CD)/5–flucytosine (5-FC), and rabbit carboxylesterase (rCE)/irinotecan (Banerjee et al., 2021). Among these, a classic example of using OV vectors to deliver conditionally toxic enzymes is Toca 511, an oncolytic **retrovirus** encoding CD (Mitchell et al., 2017).

#### Expression of E-Cadherin

Improving viral infectivity is also a good strategy for increasing the tumor-killing effect of OVs. Natural killer (NK) cells, as innate immune cells that can rapidly clear viruses, significantly limit the replication of OVs in tumors. KLRG1 is an inhibitory receptor expressed on NK cells. E-cadherin (E-cad), a calcium-dependent cell–cell adhesion molecule, effectively inhibited the killing effect of NK cells by binding to its receptor KLRG1 (Li et al., 2009). Engineering oHSV to overexpress E-cadherin in GBM cells protected the virus from clearance by NK cells toward enhancing its antitumor effect (Xu et al., 2018).

## Bystander Effects of OVs

In addition to the direct oncolysis-induced cell killing, OVs can indirectly kill uninfected tumor cells through the “bystander effect”. The antigen released by OV after the lysis of tumor cells activates tumor antigen-specific cytotoxic T lymphocytes (CTLs). Viruses and infected tumor cells then recruit innate immune cells and release numerous cytokines. These cytokines exert immunomodulatory effects that promote the maturation of APCs and the activation of adaptive antitumor immunity (Kaufman et al., 2015). Therefore, cytokines delivered by OV vectors can enhance their antitumor effects by enhancing the bystander effect of OVs. Several viruses have been engineered to express different cytokines, such as GM-CSF, IL12, and IL15 (de Graaf et al., 2018). A clinical trial evaluating the efficacy of IL12-expressing oHSV in GBM is ongoing (Patel et al., 2016).

## OVs Targeting Glioblastoma Stromal Cells OVs Acting on ECM

Cancer-associated fibroblasts (CAFs) are activated fibroblasts that constitute the major stromal component of several types of cancer, including breast, pancreatic, and lung cancers (Ligorio et al., 2019; Zhou et al., 2022). Accumulating evidence suggests that CAFs participate in tumor progression by promoting ECM remodeling and interacting with tumor cells. Although the current understanding of CAFs or CAF-like stromal cells in GBM is extremely limited. However, existing evidence suggests that pericytes, marked by the co-expression of FAP and PDGFRβ, represent a major stromal component shared by GBM patients and mouse models (Li M. et al., 2020). In fact, the study also demonstrated the unique property of the oncolytic adenovirus ICOVIR15 carrying Δ24-E1A and RGD fibers to target GBM cells and GBM-associated stromal FAP+ cells.

The modification of the physical components of the ECM is an effective approach to increasing viral replication in GBM. Hyaluronan (HA) is the main component of ECM in GBM and is involved in tumor cell invasion and migration. Arming hyaluronidase, an enzyme that selectively dissociates HA, with OVs is an attractive approach. It has been shown that the degradation of HA can enhance the immunotherapy of GBM by oncolytic adenovirus (ICOVIR 17) by overcoming the immunosuppressive function of the GBM ECM (Kiyokawa et al., 2021). Coincidentally, an oHSV equipped with the ECM-modifying protein matrix metalloproteinase (MMP) 9 showed increased replication and enhanced therapeutic efficacy in GBM (Sette et al., 2019).

### OVs acting on the vasculature

As a highly angiogenic tumor, massive vascular growth is crucial for the progression and invasion of GBM (Hardee and Zagzag, 2012). The tumor vasculature plays a key role in oxygen and nutrient transport, immune regulation, growth, and proliferation of tumor cells. Different from the vasculature in healthy tissues, the tumor vasculature in GBM is characterized by high proliferation, abnormal vasculature, and instability, which lead to the development of hypoxic regions and frequent hemorrhaging (Carmeliet and Jain, 2000). In addition, the increased vascular permeability caused by tumor ECs overexpressing VEGF receptors is associated with edema (Jain et al., 2007).

The expression of vascular endothelial growth factor (VEGF) is upregulated in GBM. Therefore, the current mainstream anti-angiogenic drugs mainly target the VEGF-VEGFR signal transduction pathway, including monoclonal antibodies to VEGF and small molecule inhibitors of VEGF receptor (VEGFR). However, in addition to the inherent disadvantages offered by their systemic administration, anti-VEGF agents also promoted the GBM tumor escape and induced tumor invasion (Kunkel et al., 2001; Lucio-Eterovic et al., 2009). Based on their selective replication ability, OVs provide an opportunity to overcome their limitation as an effective vector for delivering antiangiogenic genes. A typical example, Ad-ΔB7-KOX (an oncolytic Adv expressing a transcriptional repressor targeting the VEGF promoter) effectively retains the tumor-selective effect of OVs and significantly inhibits VEGF in GBM, demonstrating enhanced antitumor effect and survival benefits (Kang et al., 2008). In addition, arming OVs with angiogenesis inhibitors is also suggested to be an effective approach. The rapid antiangiogenesis mediated by the oncolytic (RAMBO) virus and HSV-1-expressing angiostatin (Vstat120) effectively inhibits internal angiogenesis and significantly inhibits intracranial and subcutaneous tumor growth in mice (Hardcastle et al., 2010). IL-12 also demonstrates a strong antiangiogenic effect in the body (Tahara and Lotze, 1995), which has been proved by the *in vivo* efficacy of G47Δ-mIL12 (Cheema et al., 2013).

Furthermore, the combination of genetically engineered OVs with other anti-angiogenic therapies has demonstrated greater advantages. The conventional antiangiogenic agent increases virus distribution by reducing IV collagen in the ECM (Thaci et al., 2013), which not only enhances oncolysis but also increases the effective delivery of therapeutic genes. Antiangiogenic treatment reduces the infiltration of TAMs owing to reduced vascular permeability, which in turn protects the virus from the clearance by TAM and increases the replication and spread of OVs (Kurozumi et al., 2007; Zhang et al., 2013). More importantly, genetically engineered OVs reduce tumor invasion induced by antiangiogenic drugs and enhance synergistic antiangiogenic or antitumor effects (Zhang et al., 2012; Saha et al., 2018).

### OVs remodeling on the GBM immune microenvironment

#### OVs initiating T-cell response

Although the number of infiltrating T cells in GBM is small compared with other tumor types, existing evidence has shown that tumor T-cell infiltration is positively correlated with clinical outcomes in patients with GBM, which fully demonstrates the potential of T cells in GBM treatment (Kmiecik et al., 2013; Han et al., 2014; Li et al., 2016). In fact, an effectively antitumor immune response cannot be achieved without the participation of T cells. A large number of preclinical and clinical data indicate that OVs can effectively heat the “cold” GBM immune microenvironment through the induced inflammatory cascade and inflammatory (immunogenic) cell death to trigger T-cell response (Bartlett et al., 2013; Chiocca and Rabkin, 2014). First, OVs replicate in tumors, causing the infiltration of several innate immune cells (NK cells and macrophages) related to inflammation. Second, the pattern recognition receptors expressed by these innate immune cells can induce a Th1 immune response by recognizing the virus itself or DAMPs released by virus-lysing tumor cells. In this process, the release of IFNs helps antigen presentation and induces subsequent T cell-mediated adaptive antitumor immunity (Zhou, 2009). For example, in the preclinical GL261 mouse model, the infiltration of NK cells, CD4^+^T cells, and T-bet^+^CD8^+^T cells increased after oncolytic Adv (DNX2401) treatment (Jiang et al., 2014). Likewise, the inflammatory response to intratumoral administration of ZIKV leads to the activation of microglia and myeloid cells to enhance antigen presentation and CD8^+^T cell-mediated antitumor effect (Nair et al., 2020).

In addition, immunogenic death (ICD) induced by OVs is also related to the infiltration of T-cell response in the GBM microenvironment. The characteristics of ICD are mainly mediated by DAMPs and TAAs, including surface calreticulin (ecto-CRT) exposure, ATP secretion, and high mobility group protein B1 (HMGB1) release. Ecto-CRT exposure acts as a signal to promote phagocytosis of tumor cells, whereas HMGB1 and ATP enhance the maturation and activation of APCs. In summary, ICD induced by OVs can effectively elicit T cell-mediated antitumor immune response by presenting DAMPs and TAAs to APCs to trigger and enhance the cross-presentation of tumor antigens by APCs. In preclinical studies, NDV has been a good example of OVs inducing ICD in GBM (Koks et al., 2015). The median survival of GL261-bearing mice after NDV treatment was significantly prolonged, which was accompanied by increased IFN-γ^+^T-cell infiltration. In the animal model of CD8^+^T cell depletion, the severe weakening of these therapeutic advantages indicates that T cells play an important role in an antitumor immune response. Similarly, OVs that can induce ICD in GBM include MV and oncolytic hTERT-Adv (Ito et al., 2006; Hardcastle et al., 2017).

#### OVs block immunosuppressive cells to enhance T-cell response

Tregs are mainly lymphocytes that inhibit the antitumor T-cell response. Studies have shown that the ratio of Tregs to CD4^+^T cells in the tumor and peripheral blood of GBM patients is high, leading to the failure of the remaining CD4^+^T cells (Fecci et al., 2006). Therefore, blocking the function of Tregs is an important way to enhance antitumor T-cell immunity. In the murine GBM model, systemic administration of the anti-CD25 antibody significantly inhibits the function of Tregs, thereby enhancing the antitumor ability of CD8^+^T cells (Fecci et al., 2006). Similarly, in addition to inducing powerful antitumor T-cell immunity, OVs also remodel the immunosuppressed T-cell subsets. For instance, intratumorally administration of DNX2401 increases the infiltration of CD4^+^ and CD8^+^T cells in the tumor while significantly reducing the number of Tregs (Qiao et al., 2015). Subsequent mechanism studies have indicated that DNX2401 remodels Tregs from an immunosuppressed state to an immunostimulatory state by downregulating the expression of the gene encoding IDO (Qiao et al., 2015).

MDSCs are congenital myeloid-derived immunosuppressive cells. At present, the important role of MDSCs in the progression of GBM has been widely recognized, and their accumulation is often related to the poor prognosis of GBM patients (Alban et al., 2018; De Leo et al., 2020). MDSCs produce large amounts of arginase and NO to inhibit T-cell function through a variety of mechanisms. Both preclinical and clinical results have indicated that the depletion of MDSCs in GBM murine models and GBM patients can lead to increased infiltration of activated T cells (Otvos et al., 2016; Peereboom et al., 2019). Similarly, the accumulation of MDSCs was significantly reduced in GL261 *in situ* mouse models treated with NDV, accompanied by increased infiltration of IFN-γ^+^T cells (Koks et al., 2015). This is sufficient to demonstrate that OVs can activate the immunosuppressive GBM microenvironment by remodeling immunosuppressive cells.

#### Engineered OVs enhancing T-cell response

OVs can be designed to deliver immunomodulatory factors selectively expressed in the tumor cells to enhance the antitumor immune response. Cytokines are one of the more common immune regulatory factors that can trigger powerful antitumor immunity by recruiting and activating T cells. Engineering OVs to express IL-2, IL-12, and TNF have demonstrated therapeutic effects in other mouse tumor models (Tähtinen et al., 2016; Weiss et al., 2020). Moreover, the engineered OVs—talimogene laherparepvec—was approved by the FDA in 2015 as the first OV drug for the treatment of melanoma, which is an HSV-1 inserted into the human granulocyte-macrophage colony-stimulating factor (GM-CSF) gene toward enhancing the recruitment and activation of DCs. Oncolytic HSV carrying mIL-12 has also demonstrated a survival advantage in the mouse GBM model; the results clearly indicated that this survival advantage is attributable to the increased infiltration of cytotoxic T cells (Cheema et al., 2013; Alessandrini et al., 2019). A recent study utilized the combination therapy of an IL15 super-cytokine agonist-expressing OV with EGFR-CAR NK cell therapy to significantly improve the antitumor efficacy of GBM (Ma et al., 2021).

Full activation of T cells requires the participation of costimulatory molecules. Therefore, enhancing the T cell attack against tumors by targeting costimulatory pathways is an attractive approach for anti-GBM immunotherapy. Making OVs express T-cell costimulatory molecules, for example, through the interaction of the costimulatory molecule OX40 with its ligand OX40L, can effectively stimulate T-cell activation. Delta-24-RGDOX (oncolytic Adv-expressing OX40L) can not only recruit lymphocytes to the tumor site through ICD, like DNX2401, but the activated lymphocytes are specific to tumor-associated antigens (Jiang et al., 2017).

#### OVs shifting M2-TAM into antitumor M1-TAM

Although the antitumor activity induced by OVs is mainly mediated by T-cell response, the effective presentation of tumor antigens also plays an important role in this process, especially in the GBM microenvironment where TAM limits lymphocyte immune surveillance. The functions of M1-TAM and M2-TAM are at two extremes. M1 polarization, which mainly occurs in the initial stage of an inflammatory response, improves T-cell infiltration and induces a subsequent adaptive antitumor response, whereas M2-TAM in GBM inhibits antigen presentation and adaptive immunity, and is associated with tumor development and worsening patient prognosis (da Fonseca and Badie, 2013; Mantovani and Allavena, 2015), Therefore, the conversion of tumor-promoting M2-TAM to tumor-killing M1-TAM is being developed as a potential anti-GBM strategy. Studies have shown that blocking the expression of CD47 on the surface of tumor cells or the CCR5 receptor expressed by the microglia in GBM can both effectively increase the ratio of M1/M2 and enhance tumor phagocytosis (Miyauchi et al., 2016; Zhang et al., 2016). OVs can enhance T cell-mediated antitumor immunity by inducing the activation of TAMs and promoting antigen presentation. In addition, IL-12 is a powerful inducer of IFN-γ and can promote the polarization of M1-TAM. Hence, genetically engineered HSV-IL12 shows a great synergistic effect in triple combination therapy with anti-CTLA4 and anti-PD-1 antibodies. Wouter et al. showed that the transfer of M2-TAM to M1-TAM in human GBM is caused by the synergistic effects of soluble factors produced by virus-infected tumor cells and the viral particles in oncolytic virotherapy for GBM (van den Bossche et al., 2018). All these findings suggest that TAM polarization can induce a pro-inflammatory microenvironment that can prevent tumor development.

## The Current Status of Clinical Research on OVs for GBM Treatment

Presently, a diverse range of OVs against GBM has entered different clinical trial stages. The ongoing and completed clinical trials of OVs against GBM are listed in Table 2. Among these, the most notable ones include DNX2401, Toca 511, PVS-RIPO, and G207, all of which have published results from their respective phase-I clinical trials (Cloughesy et al., 2016; Desjardins et al., 2018; Lang et al., 2018; Romero, 2021). DNX2401 is a tumor-selective oncolytic Adv. According to the published phase-I clinical data, the median overall survival of GBM patients treated with DNX2401 reached 9.5 months, and 20% of these patients survived for >3 years after the treatment (Lang et al., 2018). Moreover, the tumor site after treatment exhibited inflammation and necrosis, and histopathological examination revealed CD8^+^T cell and T-bet^+^ infiltration. This observation indicated that DNX2401 could ensure tumor regression through direct oncolysis, and it could induce an antitumor immune response, which coincided with the results of the preclinical experiments (Jiang et al., 2014; Qiao et al., 2015). The combination of DNX2401 with other therapies has also entered the clinical phase I/II trials (NCT02798406, NCT02197169, and NCT01956734). Toca 511 is a retrovirus encoding CD that can selectively replicate in tumor cells to produce a CD. Subsequently, CD transforms the prodrug 5-fluorouracil (5-FC) into chemotherapeutic 5-fluorouracil (5-FU), thereby avoiding the systemic toxicity of 5-FU. In a past study, the median overall survival of GBM patients receiving this therapy reached 14.4 months, and five complete remissions were observed (Cloughesy et al., 2016). PVS-RIPO also demonstrated favorable outcomes for treatment of recurrent GBM, with overall survival of 24 months (Desjardins et al., 2018). In addition, combination therapy with pembrolizumab has presently entered phase-II clinical trials (NCT04479241). Modified neurotropic oncolytic HSV-1 G207 has been reported to showcase durable antitumor properties in the treatment of pediatric malignant high-grade glioma (NCT00157703).

**Table 2:** Ongoing or completed clinical trials of OVs against GBM.

**Virus type**	**Mechanisms of tumor selectivity and antitumor effects**	**Oncolytic virus**	**Virus construct**	**Status/Phase**	**Administration Regimen**	**Trial No.**
Herpesvirus 1	1. HSV with TK gene deletion relies on the actively dividing cells to supply TK for republication. 2. HSV with γ34.5 deletion and UL39 mutation uses enzymes provided by actively diving tumor cells to republicated 3. HSV expressing IL-12 exerts anti-tumor effects *in vivo* through oncolysis and T cell-mediated immune effects	C134 M032 G207 rQNestin	Deletion of γ34.5 and insertion of IRS1 gene of HCMV Armed with hIL-12 Deletion of γ34.5 and lacZ insertion into the UL39 gene Deletion of UL39 and restoring one copy of ICP34.5 under nestin	Active; not recruiting; I Recruiting; I Completed; I/II Recruiting; I	IT Single IT through catheters Single IT through catheters IT after Cyclophosphamide IV 2 days	NCT03657576 NCT02062827 NCT00028158 NCT03152318
Adenovirus	1. Adv with E1B and E1A mutations target defects in p53 or Rb pathways of tumor cells 2. RGD or EGFR modification retargets GBM cells with low CAR expression 3. The anti-tumor effect *in vivo* mainly depends on oncolysis and the changes in the GBM microenvironment mediated by T cells and macrophages	DNX2401 DNX2401 DNX2401 DNX2401 DNX2240 CRAd-S-pk7	Deletion of E1B and insertion of an RGD-4C peptide motif Armed DNX2401 with OX40L Fibers were modified by incorporating polyline sequences	Completed; I/II Active; not recruiting; II Completed; I Completed; I Recruiting; I Completed; I	IT IT before pembrolizumab IV 7–9 days Single IT with or without IFN-γ injection Injection in parenchyma before TMZ orally 14 days Stereotactical injection A resection followed by injection of NSCs loaded with CRAd-S-pk7; TMZ	NCT01582516 NCT02798406 NCT02197169 NCT01956734 NCT03714334 NCT03072134
Poliovirus	1. Tropism for highly expressing CD155 receptor on tumor cells 2. HRV2 substituting IRES sequence attenuates neurotoxicity and shows better oncolytic activity	PVSRIPO	Replacing IRES of polio with HRV2	Recruiting; I Recruiting; II	Single IT IT *via* CED; pembrolizumab IV every 3 week	NCT03043391 NCT04479241
Measles virus	1. Tropism for highly expressing CD46 receptor on tumor cells 2. Oncolysis and anti-tumor immunity stimulated through ICD	MV-CEA	Expressing carcinoembryonic antigen gene (CEA)	Completed; I	Injection into resection cavity or around tumor bed or IT	NCT00390299
Parvovirus H1	1. Replicating in actively dividing cells in S-phase 2. Anti-tumor effect results from oncolytic effects and immune reaction	H-1PV	Wild-type	Completed; I/II	IT or IV 3 times and then injection into the walls of tumor resection cavity after the first IT	NCT01301430
Reovirus	1. Tropism for RAS-upregulated tumor cells 2. Adding GM-CSF is to enhance the antitumor immune response	REOLYSIN	Wild-type	Completed; I Active; not recruiting; I	IT IV before Sargramostim SC	NCT00528684 NCT02444546
Vaccinia virus	1. TK gene deletion relies on the actively dividing cells to supply TK for republication 2. Adding GM-CSF is to enhance the antitumor immune response 3. The inserted FCU1 gene together with the prodrug 5-Fc inhibits tumor growth	TG6002	Deletion of TK and RR and expressing cytosine deaminase	Recruiting; I/II	IV 3 times, followed 5-FC orally 4 times per day	NCT03294486
Retrovirus	1. Delivery therapeutic genes	Toca 511	Encoding CD	Completed; I	Injected into the resection cavity, followed 5-FC orally	NCT01470794

Search result from ClinicalTrials.gov. Abbreviations: IT, intratumorally; IV, intravenous; IA, intraarterial; IRS-1, insulin receptor substrate-1; HRV2, human rhinovirus 2; IRES, internal ribosomal entry site; 5-FC, 5-fluorocytosine; RR, ribonucleotide reductase gene; CD, cytosine deaminase; CED, convection enhanced delivery.

## Challenges of Oncolytic Virotherapy

**Figure 3:**
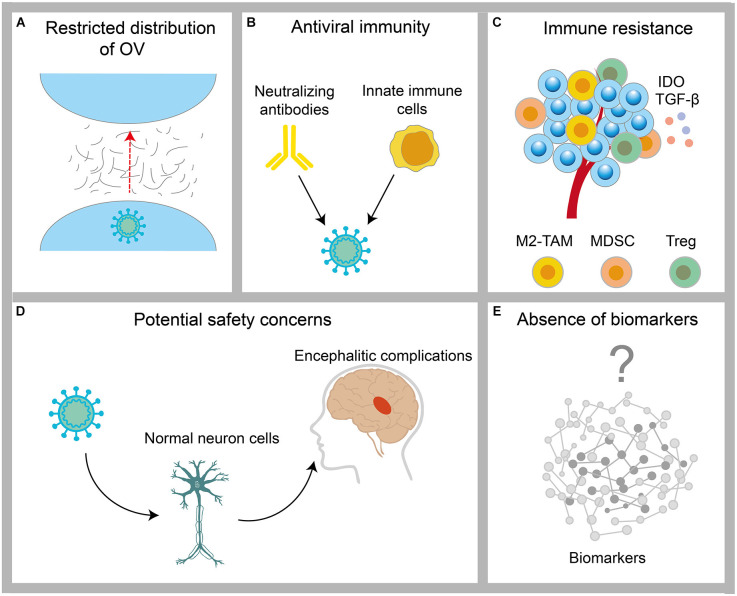
Challenges encountered by OV in the treatment of GBM. **(A)** The distribution of OVs is restricted by physical barriers, such as the tumor extracellular matrix. **(B)** Clearance of OV by host antiviral immunity. **(C)** Protection of tumor cells by immunosuppressive cells, such as M2-TAM, MDSC, and Treg in the tumor microenvironment. **(D)** The infection risk of OV to normal neuronal cells. **(E)** The lack of validated biomarkers of OVs for GBM.

Despite the existence of encouraging preliminary results, oncolytic virotherapy remains challenging for GBM (Figure 3). These potential limitations have been discussed below.

### Tumor ECM

Tumor ECM has been demonstrated in solid tumors, other than GBM, to inhibit the therapeutic effects of chemotherapy and radiotherapy through various mechanisms (Whatcott et al., 2011). Several preclinical studies have demonstrated that the physical factor limiting intratumoral dissemination of OVs is primarily high interstitial fluid pressure in the tumor mass resulting from increased secretion of the ECM components, including hyaluronic acid (HA) and collagen (Wojton and Kaur, 2010). In addition, relevant literature has shown that the increased secretion of ECM components secreted by GBM cells, such as HA, fibronectin, thrombospondin, and tenascin-C, also contributes to this modification (Khoonkari et al., 2022). Although the modification of OVs to target the ECM can increase intratumoral dissemination of the virus, as described above, the potential safety and therapeutic efficacy risks exist for the modification of a viral genome.

### Host immune response

The conflict between the virus and the host immune system has always been the key to determining the antitumor ability of OVs. The obstacles to the therapeutic response of OVs are mainly antiviral immunity mediated by neutralizing antibodies *in vivo* and innate immune cells. Presently, most delivery methods of OVs focus on intratumorally delivery to maximize the biological distribution of the virus in the tumor cells as well as to avoid neutralizing the virus through systemic humoral immunity. Despite the obstacles in the intertumoral delivery of tumors in CNS, convection-enhanced delivery systems have been developed in clinical practice for the delivery of OVs, such as DNX2401 and NCT01582516. However, the innate immune cells within a tumor are present with an important barrier. The combination of cyclophosphamide (CPA), an immunosuppressant, can effectively inhibit innate immunity and thereby effectively avoid the elimination of OVs (Qiao et al., 2008). In a preclinical study, OVs exhibited significant antitumor effects through the intertumoral biotransformation of the CPA prodrug (Ichikawa et al., 2001).

### Biosecurity

OVs carry the risk of infecting normal cells. T-VEC, the first OV-engineered OV drug approved by the FDA for the treatment of melanoma, presents the potential for long-term latent infection and neurological adverse events (Harrington et al., 2017; Li L. et al., 2020). The pathogenicity and neurovirulence of OVs have been attenuated by the use of attenuated human vaccine strains, repeated passaging, or the deletion of virulence genes. For example, HSV products 1716 and G207, with *ICP34.5* deleted, exhibited acceptable safety in clinical trials (Aghi et al., 2008). However, the deletion of virulence genes is often accompanied by diminished efficacy. In addition, some OV candidates for the treatment of GBM, such as the ZIKV, are often accompanied by encephalitic complications (Chen et al., 2018).

### Bioactivity

Generally, standard methods are used to monitor the effectiveness of OVs for GBM, including imaging and biomarkers. Magnetic resonance imaging (MRI) is important for evaluating GBM owing to its high clinical diagnostic performance (Suh et al., 2019). However, the increase in tumor volume caused by immune infiltration (pseudo progression) poses a significant challenge for the accurate assessment of the disease state (Hygino da Cruz et al., 2011). In addition, different OVs enter the tumor *via* different mechanisms and killing methods; therefore, there remains a lack of validated biomarkers of OVs for GBM treatment. Significant investment in clinical trials is therefore essential to identify biomarkers related to the response of OVs for GBM treatment.

## Novel Directions for Oncolytic Virotherapy of GBM

**Figure 4:**
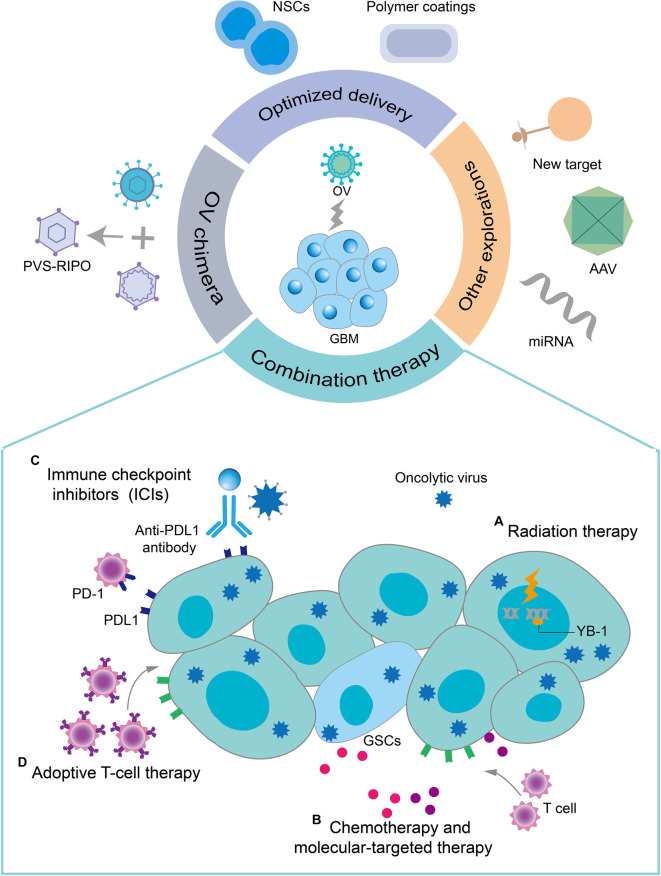
Novel directions for oncolytic virotherapy of GBM. Combination therapy with oncolytic virotherapy, optimized delivery of OVs, OV chimeras, and some other explorations provide promising therapeutic options for oncolytic virotherapy for GBM. Some common combination therapy strategies are described here. **(A)** OVs sensitize infected GBM cells to radiation therapy by preventing their DNA damage repair. RT can enhance the replication of OVs in tumor cells by modifying the gene expression in drug-resistant tumor cells. **(B)** Chemotherapy and some molecularly targeted drugs have immunomodulatory effects, which together with oncolytic virotherapy could exert a synergistic antitumor immune effect. In addition, OVs selectively induced apoptosis of TMZ-resistant GSCs by regulating the apoptosis-related signaling pathways and DNA-damage response pathways. **(C)** The upregulation of PD-1 on T cells and PD-L1 on tumor cells induced by OVs infection increased the sensitivity of GBM to immune checkpoint inhibitors (ICIs). OVs improve the effectiveness of ICIs in GBM with low T-cell infiltration by recruiting T cells and inducing an antitumor T-cell response. **(D)** The increased expression of MHC class I molecules, cell-derived damage-associated molecular patterns (DAMPs), and cytokine release resulting from OV infection enhanced the recruitment and activity of adoptive T cells to GBM.

The novel direction of oncolytic virotherapy for GBM mainly involves overcoming the abovementioned limitations by exploring different approaches (Figure 4).

### Combination therapies with oncolytic virotherapy for GBM

Similar to other cancer therapies, future GBM treatment strategies warrant a focus on the use of a combination of multiple modalities. In this section, we have briefly reviewed the rationale for using OVs as the basis for combination therapy.

#### Combination radiation therapy

Radiation therapy (RT) is an important treatment strategy for GBM. Like oncolytic therapy, RT also exerts its effect through topical application to tumors, hence the combination of the two is quite attractive. RT can enhance the replication of OVs in tumor cells by changing the gene expression in drug-resistant tumor cells. Studies have shown that RT upregulates the expression of human transcription factor YB-1 in the nucleus of GBM cells and enhances the replication of oncolytic Adv (Bieler et al., 2008). Moreover, ionizing radiation for a specific time can enhance the activity of OVs in tumors. For example, ionizing radiation can complement the deletion of the γ34.5 gene in HSV-1 by activating the p38 pathway in GBM cells (Mezhir et al., 2005). In the treatment of mice with intracranial GBM, the combination of ionizing radiation and R3616 (oncolytic HSV) showed a better therapeutic effect than monotherapy (Bradley et al., 1999). In a recent phase I clinical study on pediatric malignant glioma, after AdV-tk was applied to the surgical resection bed, RT was performed, which showed good safety and potentially significant survival results (Kieran et al., 2019).

#### Combination chemotherapy

TMZ, an oral prodrug alkylation agent, is presently the first-line clinical chemotherapy for the treatment of GBM. TMZ alkylates (methylates) the DNA to cause cell damage and eventually apoptosis. Although the introduction of TMZ in standard care was once considered a breakthrough in GBM treatment, it only prolonged the median survival of GBM patients by less than 3 months compared to RT alone (Stupp et al., 2005). In addition, tumor resistance to alkylation agents due to the MGMT DNA repair system is also a major clinical problem that remains to be solved (Hegi et al., 2005).

A growing body of preclinical data suggests that OVs are promising antitumor agents for overcoming TMZ resistance (Kanai et al., 2012; Jahan et al., 2017; Bai et al., 2018). oHSV-TRAIL (an engineered oncolytic HSV) selectively induces apoptosis of primary and recurrent TMZ-resistant GSCs by regulating apoptosis-related signaling pathways and DNA damage response pathways and prolongs the survival rate of mice bearing the same tumor cells (Jahan et al., 2017). Moreover, in the combination of NDV and TMZ, NDV enhances the antitumor effect of TMZ by inhibiting the Akt signaling pathway and activating AMPK (Bai et al., 2018). In addition to the traditional direct antitumor effects, TMZ also has immunomodulatory effects (Karachi et al., 2018). In the DNX-2401/TMZ treatment regimen for GBM, TMZ significantly promotes the effective recognition of tumor cells by CD8^+^T cells (Kleijn et al., 2017).

#### Combination immunotherapy

Cancer immunotherapy uses the body’s own innate and adaptive immune system to attack and destroy tumors. Nowadays, immunotherapy has been firmly established in the treatment of various malignant tumors but the efficacy of immunotherapy for GBM is not ideal. Monotherapy using immune checkpoint inhibitors (ICIs) has shown limited curative effect in preclinical and clinical trials, whereas the combined use of anti-PD-1 and anti-CTLA-4 has shown severe adverse reactions in clinical trials, despite encouraging results in preclinical trials (Preusser et al., 2015; Reardon et al., 2016; Omuro et al., 2018). Ongoing clinical trials of chimeric antigen receptor T (CAR-T) cell therapy and vaccination strategies have culminated in disappointing results (Brown et al., 2016; Rampling et al., 2016; O’Rourke et al., 2017). Effective immunotherapy relies on the immune status within the tumor microenvironment. In general, tumors with low TIL infiltration are less responsive to immunotherapy (Topalian et al., 2016). Therefore, the immunosuppressive GBM microenvironment is the main reason for the limited efficacy of immunotherapy in GBM, especially for T cells, including immunosuppressive immune cells (Tregs, MDSCs, and TAMs), tumor cell-derived inhibitory cytokines, and the upregulated expression of immune checkpoint receptors on T cells and PD-L1 on tumor cells.

The combination of OVs and ICI is attractive. On one hand, OVs can increase the effectiveness of ICI in GBM with low T-cell infiltration by recruiting T cells and inducing antitumor T-cell response. On the other hand, the upregulation of PD-1 on T cells and PD-L1 on tumor cells induced by OVs through an inflammatory response also increases the sensitivity of GBM to ICIs (Jiang et al., 2017; Samson et al., 2018). More importantly, the synergy of the combination of the two in transforming cold tumors into hot tumors is in line with the current strategy of combined immunotherapy (Lim et al., 2018). At present, multiple preclinical results have shown the significant curative effect of the combination of ICI and oncolytic virotherapy. For instance, ZIKV, MV, reovirus, and VSV (respectively armed with HIF-2α, Sox-10, c-Myc, tyrosinase-related protein 1) and oncolytic adenovirus (expressing hyaluronidase), respectively, combined with the anti-PD-1 antibody show better therapeutic effects than single therapy in GL261 or CT2A orthotopic mice models (Cockle et al., 2016; Hardcastle et al., 2017; Samson et al., 2018; Nair et al., 2020; Kiyokawa et al., 2021). The combined therapy of oncolytic Adv Delta-24-RGDOX and anti-PD-L1 antibody also shows the same superiority (Jiang et al., 2017). In addition, the G47Δ-mIL12 joined with two types of ICI cured the vast majority of mice in the two mouse models, while potentially reducing the clinical toxicity of anti-CTLA-4 and anti-PD-1 antibody combination (Saha et al., 2017).

Based on the above findings, the combination of OVs and adoptive T-cell therapy (ACT) is a promising combination therapy for GBM. Generally, the continued effective response of ACT depends on the delivery and survival of donor T cells to the tumor site (Lichty et al., 2014). Therefore, OVs can better promote the response of ACT to GBM by regulating the local tumor microenvironment. In addition to recruiting large amounts of adoptive T cells to infiltrate tumor sites, OVs can also address the obstacles encountered by adoptive T cells by regulating the immunosuppressive tumor microenvironment (Bagley et al., 2018). Moreover, the release of pathogen-associated molecular patterns and TAAs caused by the selective killing of tumor cells by OVs further enhances the function of effector T cells (Tähtinen et al., 2015). It is also possible to further improve ACT by loading OVs with other potent therapeutic genes. In one study, oncolytic Adv equipped with IL-7 effectively improved the efficacy of B7H3-CAR-T in the treatment of GBM by enhancing T-cell activation signals (Huang et al., 2021). Similarly, in another preclinical study, Ad5Δ24 loaded with two immunomodulatory molecules, chemokine CCL5 receptor and IL-15, combined with CAR-T therapy significantly improved the survival rate of neuroblastoma-bearing mice (Nishio and Dotti, 2015).

#### Combination molecular-targeted therapy

GBM has undergone comprehensive molecular analyses (Verhaak et al., 2010; Ceccarelli et al., 2016). Correlation analysis suggests that most subtypes of GBM have abnormal signaling pathways, such as mitogen-activated protein kinase, phosphoinositide 3 kinase (PI3K), and receptor tyrosine kinase signaling pathways that regulate tumor cell growth and the p53 tumor suppressor pathway that regulates tumor cell cycle and DNA repair. Molecular-targeted therapy can effectively inhibit these abnormal pathways in GBM. Recently, the combination of OVs with molecular-targeted drugs has also become popular for GBM treatment. For example, the combination of a novel oncolytic HSV (MG18L) with a PI3K/Akt inhibitor increases the survival rate of bearing-GBM mice by enhancing the induction of GSC apoptosis (Kanai et al., 2011). Similarly, in the 005 mouse GSC model, after the combined use of G47Δ-mIL12 and the protein tyrosine kinase inhibitor axitinib, increased anti-GBM activity was observed than that with monotherapy (Saha et al., 2018). Axitinib regulates the tumor microenvironment and improves the immunotherapy outcome (Du Four et al., 2015). Therefore, whether the combination of the two can promote the antitumor effect of OVs to exert a stronger effect is not known, but enhanced anti-GBM activity is certainly T-cell-dependent. The presence of BBB is also a problem for the delivery of small-molecule inhibitors to the CNS. Studies have shown that OVs can promote the penetration and distribution of trametinib in the brain, thereby effectively enhancing the therapeutic effect (Yoo et al., 2019). Except for small molecular inhibitors, the combination of OVs and monoclonal antibodies targeting the VEGF–VEGFR pathway is also under active exploration. RAMBO, an engineered oncolytic HSV-1, combined with bevacizumab showed good anti-GBM activity and effectively inhibited the tumor invasion induced by bevacizumab (Tomita et al., 2019).

### Optimized delivery methods

#### Cell-based delivery vehicles

The use of optimized delivery modes for OVs is a new strategy for protecting OVs from neutralizing antibodies and reducing toxicity. Using tumor-homing cells [such as mesenchymal stem cells (MSCs) and neural stem cells (NSCs) as vectors is the most promising delivery strategy currently under investigation]. These cells not only deliver OVs to the tumor site but also protect OVs from the neutralizing antibodies present in the blood. Preclinical studies have demonstrated the enhanced antitumor efficacy of oncolytic Adv using MSCs and NSCs as cell vectors in an *in situ* mouse model of GBM (Yong et al., 2009; Ahmed et al., 2011). A phase I clinical analysis of HSV-1 (M032) using NSCs as cell carriers for the treatment of recurrent GBM is already underway (NCT02062827). Furthermore, phase-I safety analysis of intratumoral injection of neural stem cell-loaded CRAd-S-pk7 in combination with the standard-of-care chemoradiotherapy in patients with newly diagnosed high-grade glioma is ongoing (NCT03072134).

#### Coating OVs with polymer

The use of biocompatible polymers to protect the virus from antibody binding and interaction with other blood components is an effective approach to improve the blood flow stability and tumor accumulation during the intravenous delivery of OVs (Cattaneo et al., 2008). The most widely applied approach is the use of polymer coatings to coat oncolytic adenoviruses to protect them from neutralizing antibodies, a process also known as “polymer stealth”. However, the use of some polymers, such as polyethylene glycol (PEG) and poly-N-(2-hydroxypropyl) methacrylamide (polyHPMA), effectively shield the medium *via* amine-mediated covalent bonding and antibody-binding sites as well as reduce CAR-mediated endocytosis, which lowers the transduction efficiency (Wortmann et al., 2008). Therefore, methods to increase tumor selectivities, such as conjugation of the polymers to specific tumor-targeting moieties and the use of polymers that are selectively degraded in the tumor microenvironment, are being pursued (Moon et al., 2015; Garofalo et al., 2021). Although this strategy is currently less applied for OVs in the GBM field, it provides an idea for intravenously delivering OVs to cross the BBB.

#### OV chimera

To facilitate clinical translation, several genetic engineering strategies have been applied to OVs to produce the optimal OVs vector platforms, which can provide potent tumor-selective cytotoxicity and intratumoral cytotoxicity while exhibiting enhanced safety and immune-stimulatory effect. However, it is difficult for a single OVs vector to meet the above-mentioned requirements simultaneously. Therefore, viral chimeras provide opportunities for oncolytic virotherapy and other virotherapy. There is a summary of the literature on viral chimeras as an effective tool for genetically engineered viral therapy (Kaufmann and Nettelbeck, 2012). Currently, the most promising chimeric OVs is PVS-RIPO, which has entered clinical phase-II trials for the treatment of recurrent glioblastoma multiforme. This virus is based on Sabin, an attenuated poliovirus type 1 (PV) vaccine strain. Past studies have demonstrated that oncolytic PV can effectively induce the regression of GBM and generate strong antitumor immune responses in mice, albeit it has strong neurotoxicity (Gromeier et al., 2000). Therefore, exchanging its internal ribosome entry site (IRES) with the IRES of human rhinovirus 2 led to attenuated neurovirulence. Another example of the treatment of GBM is the oncolytic HSV/human cytomegalovirus chimera HSV-1Δγ 1 34.5/HCMV (Shah et al., 2007).

#### OVs armed with microRNA

The discovery of tissue-specific expression of cellular microRNAs provides a new research approach for the development of oncolytic viral vectors with enhanced tumor targeting ability and tissue tropism. MicroRNAs belong to a class of single-stranded, endogenous, post-transcriptional regulatory small molecules with important regulatory roles in cell proliferation, cell differentiation, apoptosis, and tumorigenesis through binding with the 3’-untranslated region (UTR) of targeted mRNAs toward promoting mRNA cleavage or repressing the gene expression at the post-transcriptional level. Downregulation of endogenous cellular miRNAs in tumor cells is a hallmark of cancer cells. Recent studies have demonstrated that miR7 is downregulated in GBM (Webster et al., 2009). Accordingly, an MV that expresses the target site of microRNA-7 in the 3’-untranslated region of the viral fusion gene has been designed for GBM studies (Leber et al., 2011). In addition, another study reported that wild-type HSV incorporating the miR-124 recognition element in *ICP4* did not replicate in the normal brain tissues (Mazzacurati et al., 2015). In conclusion, the application of microRNA can have a remarkable effect on enhancing the safety and tumor-targeting ability of OV vectors.

### Searching for potential biomarkers of OVs against GBM

No reliable predictive biomarkers have yet been identified to correlate with the treatment response to OVs in GBM. OVs used to treat GBM enter the tumor cells through different mechanisms and induce killings by different mechanisms. As more and more OVs enter the stage of clinical surveillance, comprehension of the pathways and molecules related to the sensitivity and resistance of OVs can offer several opportunities for the prediction of potential biomarkers. Although a wealth of *in vivo* and *in vitro* data provided relevant and valuable information, the search for suitable biomarkers continues to require extensive validation. With the rapid development in this field in the past decade, the application of proteomics is expected to improve the efficiency of this work (Chen et al., 2021). First, these proteins are widely found in the blood and cerebrospinal fluid. Recent studies have reported that proteomics combined with other approaches can be applied to evaluate pediatric brain cancer from the perspective of exploring new biomarkers (Petralia et al., 2020). Second, as special immunotherapy, the immunostimulatory effect of OVs has been gradually recognized as the dominant factor influencing the antitumor effect *in vivo*. A past study demonstrated that DNX2401-induced changes in inflammatory cytokines in the CSF of patients with GBM may be the key to therapeutic success, suggesting that cytokines may serve as a biomarker for oncolytic Adv to treat GBM (van den Bossche et al., 2018). Therefore, immuno-based proteomics may be a valuable platform for the discovery of reliable biomarkers.

### New targets for oncolytic virotherapy against GBM

OVs can be flexibly loaded with effective therapeutic genes. Therefore, OVs can often take advantage of emerging targets or therapeutics. Currently, the potential therapeutic targets for GBM are mainly GSCs. Drug-resistant GSCs are the main factors contributing to the recurrence of GBM. Recent studies have revealed their ability to induce self-renewal and M2-like polarization in TAMs (Yin et al., 2020). A recent synthetic lethal strategy for GBM employed combined inhibition of autophagy and proteasome, which increased the cytotoxicity of PTEN-expressing GSCs and specifically increased the cell death markers in 3D GBM organoids (Benitez et al., 2021). Another emerging target against GSCs is calcium/calmodulin-dependent protein kinase II (CaMKII). There is supporting evidence signifying that CaMKII is involved in the survival, proliferation, and maintenance of GSCs (Han et al., 2020). Furthermore, a past study proposed a novel combination therapy targeting CaMKII for eradicating synthetic lethality in GSCs (Han et al., 2022).

### Exploration of the oncolytic potential of AAV in GBM therapy

AAV is a single-stranded linear DNA-deficient virus widely used as a non-replicating viral vector for GBM gene therapy. AAV vectors possess the characteristics of relatively low cytotoxicity, poor immunogenicity, broad tissue tropism, and long-term stable transgene expression (Xu et al., 2021). AAV is widely applied in the treatment of GBM by carrying effective therapeutic genes, including anti-angiogenic genes, cytotoxicity or suicide genes, and immune-stimulating genes (Mormino and Garofalo, 2022). Recently, an AAV-mediated CRISPR screening technique was applied to identify the functional inhibitors of GBM in the native microenvironment of the mouse brain (Chow et al., 2017). In addition, a recent study gained attention wherein Sleeping Beauty (SB) transposon-nested AAV vector-mediated CRISPR screening could successfully screen T cell membrane proteins in a mouse model of GBM and enhanced the engineered CAR-T Effector killing of human GBM cells (Ye et al., 2019). However, in contrast to OV, AAV has demonstrated no direct oncolytic effect and cannot replicate independently. Moreover, its replication and cytolytic functions can only be performed in the presence of a helper virus (Meier et al., 2020). Previous reports have indicated that AAV replication may occur in cells exposed to various genotoxic or cytostatic conditions (Nicolas et al., 2012). Therefore, creating conditions to aid AAV replication by inducing transformed cell lines may stimulate its independent replication. Notably, a past study demonstrated that AAV enhanced Ad-mediated cytotoxicity and oncolytic potency, which opens the possibility of applying AAV in oncolytic virotherapy (Laborda et al., 2013).

## Conclusions

As a promising cancer therapeutic agent, OVs have demonstrated an impressive curative effect on GBM. OVs act on the GBM microenvironment through multiple mechanisms: the direct killing of GBM tumor cells, unique selective killing of GSCs, disrupting tumor angiogenesis, and remodeling of the immunosuppressive microenvironment. Meanwhile, engineering these viruses to express other therapeutic loads also makes OVs a highly flexible and potentially promising platform. Moreover, the combination of OVs, immunotherapy, and molecular-targeted therapy has exhibited a significant synergistic outcome. Notably, when combining with other therapies or using its carrier characteristics, the selection of the best virus, the best dose, the route of administration, and the schedule need further research. Overall, oncolytic virotherapy is one of the most compelling approaches to GBM treatment.

## Author Contributions

ZQ conceptualized, researched, and wrote the manuscript including figures and tables. XL participated in the revision of the manuscript and the collection of related literature. PC and JL provided constructive guidance and critical advice on the framework of this review. All authors contributed to the article and approved the submitted version.

## Funding

This work was supported by the National Science and Technology Major Projects of New Drugs (2018ZX09201018-013), the National Science and Technology Major Project for Infectious Diseases Control (2017ZX10203206-004), the National Natural Science Foundation of China (81101728).

## References

[B1] Abbott N. J. (2013). Blood-brain barrier structure and function and the challenges for CNS drug delivery. J. Inherit. Metab. Dis. 36, 437–449. 10.1007/s10545-013-9608-0 23609350

[B2] Ady J. W. Heffner J. Klein E. Fong Y. (2014). Oncolytic viral therapy for pancreatic cancer: current research and future directions. Oncolytic Virother. 3, 35–46. 10.2147/OV.S53858 27512661 PMC4918362

[B3] Aghi M. Visted T. Depinho R. A. Chiocca E. A. (2008). Oncolytic herpes virus with defective ICP6 specifically replicates in quiescent cells with homozygous genetic mutations in p16. Oncogene 27, 4249–4254. 10.1038/onc.2008.53 18345032 PMC7100519

[B4] Ahmad G. Amiji M. M. (2017). Cancer stem cell-targeted therapeutics and delivery strategies. Expert Opin. Drug Deliv. 14, 997–1008. 10.1080/17425247.2017.1263615 27866420

[B5] Ahmed A. U. Thaci B. Alexiades N. G. Han Y. Qian S. Liu F.. (2011). Neural stem cell-based cell carriers enhance therapeutic efficacy of an oncolytic adenovirus in an orthotopic mouse model of human glioblastoma. Mol. Ther. 19, 1714–1726. 10.1038/mt.2011.100 21629227 PMC3182345

[B6] Alban T. J. Alvarado A. G. Sorensen M. D. Bayik D. Volovetz J. Serbinowski E.. (2018). Global immune fingerprinting in glioblastoma patient peripheral blood reveals immune-suppression signatures associated with prognosis. JCI Insight 3:e122264. 10.1172/jci.insight.122264 30385717 PMC6238746

[B7] Alessandrini F. Menotti L. Avitabile E. Appolloni I. Ceresa D. Marubbi D.. (2019). Eradication of glioblastoma by immuno-virotherapy with a retargeted oncolytic HSV in a preclinical model. Oncogene 38, 4467–4479. 10.1038/s41388-019-0737-2 30755732

[B8] Allen C. Vongpunsawad S. Nakamura T. James C. D. Schroeder M. Cattaneo R.. (2006). Retargeted oncolytic measles strains entering via the EGFRvIII receptor maintain significant antitumor activity against gliomas with increased tumor specificity. Cancer Res. 66, 11840–11850. 10.1158/0008-5472.CAN-06-1200 17178881

[B9] Anderson B. D. Nakamura T. Russell S. J. Peng K. W. (2004). High CD46 receptor density determines preferential killing of tumor cells by oncolytic measles virus. Cancer Res. 64, 4919–4926. 10.1158/0008-5472.CAN-04-0884 15256464

[B10] Angelova A. L. Barf M. Geletneky K. Unterberg A. Rommelaere J. (2017). Immunotherapeutic potential of oncolytic H-1 parvovirus: hints of glioblastoma microenvironment conversion towards immunogenicity. Viruses 9:382. 10.3390/v9120382 29244745 PMC5744156

[B11] Aref S. Bailey K. Fielding A. (2016). Measles to the rescue: a review of oncolytic measles virus. Viruses 8:294. 10.3390/v8100294 27782084 PMC5086626

[B12] Bagley S. J. Desai A. S. Linette G. P. June C. H. O’Rourke D. M. (2018). CAR T-cell therapy for glioblastoma: recent clinical advances and future challenges. Neuro Oncol. 20, 1429–1438. 10.1093/neuonc/noy032 29509936 PMC6176794

[B13] Bai Y. Chen Y. Hong X. Liu X. Su X. Li S.. (2018). Newcastle disease virus enhances the growth-inhibiting and proapoptotic effects of temozolomide on glioblastoma cells *in vitro* and *in vivo*. Sci. Rep. 8:11470. 10.1038/s41598-018-29929-y 30065314 PMC6068118

[B14] Banerjee K. Núñez F. J. Haase S. McClellan B. L. Faisal S. M. Carney S. V.. (2021). Current approaches for glioma Gene Ther. and virotherapy. Front. Mol. Neurosci. 14:621831. 10.3389/fnmol.2021.621831 33790740 PMC8006286

[B15] Bartlett D. L. Liu Z. Sathaiah M. Ravindranathan R. Guo Z. He Y.. (2013). Oncolytic viruses as therapeutic cancer vaccines. Mol. Cancer 12:103. 10.1186/1476-4598-12-103 24020520 PMC3847443

[B16] Benitez J. A. Finlay D. Castanza A. Parisian A. D. Ma J. Longobardi C.. (2021). PTEN deficiency leads to proteasome addiction: a novel vulnerability in glioblastoma. Neuro Oncol. 23, 1072–1086. 10.1093/neuonc/noab001 33428749 PMC8661409

[B17] Bieler A. Mantwill K. Holzmüller R. Jürchott K. Kaszubiak A. Stärk S.. (2008). Impact of radiation therapy on the oncolytic adenovirus dl520: implications on the treatment of glioblastoma. Radiother. Oncol. 86, 419–427. 10.1016/j.radonc.2007.10.009 17967494

[B18] Boviatsis E. J. Scharf J. M. Chase M. Harrington K. Kowall N. W. Breakefield X. O.. (1994). Antitumor activity and reporter gene transfer into rat brain neoplasms inoculated with herpes simplex virus vectors defective in thymidine kinase or ribonucleotide reductase. Gene Ther. 1, 323–331. 7584098

[B19] Bradley J. D. Kataoka Y. Advani S. Chung S. M. Arani R. B. Gillespie G. Y.. (1999). Ionizing radiation improves survival in mice bearing intracranial high-grade gliomas injected with genetically modified herpes simplex virus. Clin. Cancer Res. 5, 1517–1522. 10389941

[B20] Brown C. E. Alizadeh D. Starr R. Weng L. Wagner J. R. Naranjo A.. (2016). Regression of glioblastoma after chimeric antigen receptor T-cell therapy. N. Engl. J. Med. 375, 2561–2569. 10.1056/NEJMoa1610497 28029927 PMC5390684

[B21] Carmeliet P. Jain R. K. (2000). Angiogenesis in cancer and other diseases. Nature 407, 249–257. 10.1038/35025220 11001068

[B22] Cattaneo R. Miest T. Shashkova E. V. Barry M. A. (2008). Reprogrammed viruses as cancer therapeutics: targeted, armed and shielded. Nat. Rev. Microbiol. 6, 529–540. 10.1038/nrmicro1927 18552863 PMC3947522

[B23] Ceccarelli M. Barthel F. P. Malta T. M. Sabedot T. S. Salama S. R. Murray B. A.. (2016). Molecular profiling reveals biologically discrete subsets and pathways of progression in diffuse glioma. Cell 164, 550–563. 10.1016/j.cell.2015.12.028 26824661 PMC4754110

[B24] Cheema T. A. Wakimoto H. Fecci P. E. Ning J. Kuroda T. Jeyaretna D. S.. (2013). Multifaceted oncolytic virus therapy for glioblastoma in an immunocompetent cancer stem cell model. Proc. Natl. Acad. Sci. U S A 110, 12006–12011. 10.1073/pnas.1307935110 23754388 PMC3718117

[B25] Chen L. Qin D. Guo X. Wang Q. Li J. (2021). Putting proteomics into immunotherapy for glioblastoma. Front. Immunol. 12:593255. 10.3389/fimmu.2021.593255 33708196 PMC7940695

[B26] Chen Q. Wu J. Ye Q. Ma F. Zhu Q. Wu Y.. (2018). Treatment of human glioblastoma with a live attenuated zika virus vaccine candidate. mBio 9, e01683–e01718. 10.1128/mBio.01683-18 30228241 PMC6143740

[B27] Chiocca E. A. Abbed K. M. Tatter S. Louis D. N. Hochberg F. H. Barker F.. (2004). A phase I open-label, dose-escalation, multi-institutional trial of injection with an E1B-Attenuated adenovirus, ONYX-015, into the peritumoral region of recurrent malignant gliomas, in the adjuvant setting. Mol. Ther. 10, 958–966. 10.1016/j.ymthe.2004.07.021 15509513

[B28] Chiocca E. A. Rabkin S. D. (2014). Oncolytic viruses and their application to cancer immunotherapy. Cancer Immunol. Res. 2, 295–300. 10.1158/2326-6066.CIR-14-0015 24764576 PMC4303349

[B29] Chiocca E. A. Nassiri F. Wang J. Peruzzi P. Zadeh G. (2019). Viral and other therapies for recurrent glioblastoma: is a 24-month durable response unusual? Neuro Oncol. 21, 14–25. 10.1093/neuonc/noy170 30346600 PMC6303472

[B30] Chow R. D. Guzman C. D. Wang G. Schmidt F. Youngblood M. W. Ye L.. (2017). AAV-mediated direct *in vivo* CRISPR screen identifies functional suppressors in glioblastoma. Nat. Neurosci. 20, 1329–1341. 10.1038/nn.4620 28805815 PMC5614841

[B31] Cloughesy T. F. Landolfi J. Hogan D. J. Bloomfield S. Carter B. Chen C. C.. (2016). Phase 1 trial of vocimagene amiretrorepvec and 5-fluorocytosine for recurrent high-grade glioma. Sci. Transl. Med. 8:341ra375. 10.1126/scitranslmed.aad9784 27252174 PMC6707068

[B32] Cockle J. V. Rajani K. Zaidi S. Kottke T. Thompson J. Diaz R. M.. (2016). Combination viroimmunotherapy with checkpoint inhibition to treat glioma, based on location-specific tumor profiling. Neuro Oncol. 18, 518–527. 10.1093/neuonc/nov173 26409567 PMC4799678

[B33] da Fonseca A. C. Badie B. (2013). Microglia and macrophages in malignant gliomas: recent discoveries and implications for promising therapies. Clin. Dev. Immunol. 2013:264124. 10.1155/2013/264124 23864876 PMC3707269

[B34] Davis M. E. (2016). Glioblastoma: overview of disease and treatment. Clin. J. Oncol. Nurs. 20, S2–S8. 10.1188/16.CJON.S1.2-8 27668386 PMC5123811

[B35] de Graaf J. F. de Vor L. Fouchier R. A. M. van den Hoogen B. G. (2018). Armed oncolytic viruses: a kick-start for anti-tumor immunity. Cytokine Growth Factor Rev. 41, 28–39. 10.1016/j.cytogfr.2018.03.006 29576283 PMC7108398

[B36] De Leo A. Ugolini A. Veglia F. (2020). Myeloid cells in glioblastoma microenvironment. Cells 10:18. 10.3390/cells10010018 33374253 PMC7824606

[B37] Delgado-López P. D. Corrales-García E. M. (2016). Survival in glioblastoma: a review on the impact of treatment modalities. Clin. Transl. Oncol. 18, 1062–1071. 10.1007/s12094-016-1497-x 26960561

[B38] Desjardins A. Gromeier M. Herndon J. E. Beaubier N. Bolognesi D. P. Friedman A. H.. (2018). Recurrent glioblastoma treated with recombinant poliovirus. N. Engl. J. Med. 379, 150–161. 10.1056/NEJMoa1716435 29943666 PMC6065102

[B39] Di Piazza M. Mader C. Geletneky K. Herrero Y. C. M. Weber E. Schlehofer J.. (2007). Cytosolic activation of cathepsins mediates parvovirus H-1-induced killing of cisplatin and TRAIL-resistant glioma cells. J. Virol. 81, 4186–4198. 10.1128/JVI.02601-06 17287256 PMC1866092

[B40] Diaz R. J. Ali S. Qadir M. G. De La Fuente M. I. Ivan M. E. Komotar R. J.. (2017). The role of bevacizumab in the treatment of glioblastoma. J. Neurooncol. 133, 455–467. 10.1007/s11060-017-2477-x 28527008

[B41] Du Four S. Maenhout S. K. De Pierre K. Renmans D. Niclou S. P. Thielemans K.. (2015). Axitinib increases the infiltration of immune cells and reduces the suppressive capacity of monocytic MDSCs in an intracranial mouse melanoma model. Oncoimmunology 4:e998107. 10.1080/2162402X.2014.998107 26137411 PMC4485747

[B42] Duebgen M. Martinez-Quintanilla J. Tamura K. Hingtgen S. Redjal N. Wakimoto H.. (2014). Stem cells loaded with multimechanistic oncolytic herpes simplex virus variants for brain tumor therapy. J. Natl. Cancer Inst. 106:dju090. 10.1093/jnci/dju090 24838834

[B43] Eichler A. F. Chung E. Kodack D. P. Loeffler J. S. Fukumura D. Jain R. K.. (2011). The biology of brain metastases-translation to new therapies. Nat. Rev. Clin. Oncol. 8, 344–356. 10.1038/nrclinonc.2011.58 21487419 PMC3259742

[B44] Estevez-Ordonez D. Chagoya G. Salehani A. Atchley T. J. Laskay N. M. B. Parr M. S.. (2021). Immunovirotherapy for the treatment of glioblastoma and other malignant gliomas. Neurosurg. Clin. N. Am. 32, 265–281. 10.1016/j.nec.2020.12.008 33781507 PMC8519502

[B45] Fan X. Lu H. Cui Y. Hou X. Huang C. Liu G.. (2018). Overexpression of p53 delivered using recombinant NDV induces apoptosis in glioma cells by regulating the apoptotic signaling pathway. Exp. Ther. Med. 15, 4522–4530. 10.3892/etm.2018.5935 29731836 PMC5920899

[B46] Fecci P. E. Sweeney A. E. Grossi P. M. Nair S. K. Learn C. A. Mitchell D. A.. (2006). Systemic anti-CD25 monoclonal antibody administration safely enhances immunity in murine glioma without eliminating regulatory T cells. Clin. Cancer Res. 12, 4294–4305. 10.1158/1078-0432.CCR-06-0053 16857805

[B47] Fueyo J. Alemany R. Gomez-Manzano C. Fuller G. N. Khan A. Conrad C. A.. (2003). Preclinical characterization of the antiglioma activity of a tropism-enhanced adenovirus targeted to the retinoblastoma pathway. J. Natl. Cancer Inst. 95, 652–660. 10.1093/jnci/95.9.652 12734316

[B48] Fukuhara H. Ino Y. Todo T. (2016). Oncolytic virus therapy: a new era of cancer treatment at dawn. Cancer Sci. 107, 1373–1379. 10.1111/cas.13027 27486853 PMC5084676

[B49] Gabrilovich D. I. Nagaraj S. (2009). Myeloid-derived suppressor cells as regulators of the immune system. Nat. Rev. Immunol. 9, 162–174. 10.1038/nri2506 19197294 PMC2828349

[B50] Gambini E. Reisoli E. Appolloni I. Gatta V. Campadelli-Fiume G. Menotti L.. (2012). Replication-competent herpes simplex virus retargeted to HER2 as therapy for high-grade glioma. Mol. Ther. 20, 994–1001. 10.1038/mt.2012.22 22354378 PMC3345974

[B51] Garcia-Montojo M. Doucet-O’Hare T. Henderson L. Nath A. (2018). Human endogenous retrovirus-K (HML-2): a comprehensive review. Crit. Rev. Microbiol. 44, 715–738. 10.1080/1040841X.2018.1501345 30318978 PMC6342650

[B52] Garofalo M. Bellato F. Magliocca S. Malfanti A. Kuryk L. Rinner B.. (2021). Polymer coated oncolytic adenovirus to selectively target hepatocellular carcinoma cells. Pharmaceutics 13:949. 10.3390/pharmaceutics13070949 34202714 PMC8309094

[B53] Gatson N. N. Chiocca E. A. Kaur B. (2012). Anti-angiogenic gene therapy in the treatment of malignant gliomas. Neurosci. Lett. 527, 62–70. 10.1016/j.neulet.2012.08.001 22906922 PMC3471371

[B54] Geletneky K. Hajda J. Angelova A. L. Leuchs B. Capper D. Bartsch A. J.. (2017). Oncolytic H-1 parvovirus shows safety and signs of immunogenic activity in a first phase I/IIa glioblastoma trial. Mol. Ther. 25, 2620–2634. 10.1016/j.ymthe.2017.08.016 28967558 PMC5768665

[B55] Gong J. Mita M. M. (2014). Activated ras signaling pathways and reovirus oncolysis: an update on the mechanism of preferential reovirus replication in cancer cells. Front. Oncol. 4:167. 10.3389/fonc.2014.00167 25019061 PMC4071564

[B56] Gridley D. S. Andres M. L. Li J. Timiryasova T. Chen B. Fodor I.. (1998). Evaluation of radiation effects against C6 glioma in combination with vaccinia virus-p53 gene therapy. Int. J. Oncol. 13, 1093–1098. 10.3892/ijo.13.5.1093 9772305

[B57] Gromeier M. Lachmann S. Rosenfeld M. R. Gutin P. H. Wimmer E. (2000). Intergeneric poliovirus recombinants for the treatment of malignant glioma. Proc. Natl. Acad. Sci. U S A 97, 6803–6808. 10.1073/pnas.97.12.6803 10841575 PMC18745

[B58] Haines I. E. Gabor Miklos G. L. (2014). Bevacizumab for newly diagnosed glioblastoma. N. Engl. J. Med. 370:2048. 10.1056/NEJMc1403303 24849089

[B59] Han J. M. Kim Y. J. Jung H. J. (2022). Discovery of a new CaMKII-targeted synthetic lethal therapy against glioblastoma stem-like cells. Cancers (Basel) 14:1315. 10.3390/cancers14051315 35267623 PMC8909660

[B60] Han S. Zhang C. Li Q. Dong J. Liu Y. Huang Y.. (2014). Tumour-infiltrating CD4^+^ and CD8^+^ lymphocytes as predictors of clinical outcome in glioma. Br. J. Cancer 110, 2560–2568. 10.1038/bjc.2014.162 24691423 PMC4021514

[B61] Han X. C. Zhang Y. J. Dong X. Xing Q. Z. Li K. H. Zhang L.. (2020). Sevoflurane modulates the cancer stem cell-like properties and mitochondrial membrane potential of glioma via Ca^2+^-dependent CaMKII/JNK cascade. Life Sci. 253:117675. 10.1016/j.lfs.2020.117675 32360621

[B62] Hancock W. T. Marfel M. Bel M. (2014). Zika virus, french polynesia, south pacific, 2013. Emerg. Infect. Dis. 20:1960. 10.3201/eid2011.141380 25341051 PMC4214323

[B63] Hardcastle J. Kurozumi K. Chiocca E. A. Kaur B. (2007). Oncolytic viruses driven by tumor-specific promoters. Curr. Cancer Drug Targets 7, 181–189. 10.2174/156800907780058880 17346110

[B64] Hardcastle J. Kurozumi K. Dmitrieva N. Sayers M. P. Ahmad S. Waterman P.. (2010). Enhanced antitumor efficacy of vasculostatin (Vstat120) expressing oncolytic HSV-1. Mol. Ther. 18, 285–294. 10.1038/mt.2009.232 19844198 PMC2818668

[B65] Hardcastle J. Mills L. Malo C. S. Jin F. Kurokawa C. Geekiyanage H.. (2017). Immunovirotherapy with measles virus strains in combination with anti-PD-1 antibody blockade enhances antitumor activity in glioblastoma treatment. Neuro Oncol. 19, 493–502. 10.1093/neuonc/now179 27663389 PMC5464320

[B66] Hardee M. E. Zagzag D. (2012). Mechanisms of glioma-associated neovascularization. Am. J. Pathol. 181, 1126–1141. 10.1016/j.ajpath.2012.06.030 22858156 PMC3463636

[B67] Harrington K. J. Michielin O. Malvehy J. Pezzani Grüter I. Grove L. Frauchiger A. L.. (2017). A practical guide to the handling and administration of talimogene laherparepvec in Europe. Onco Targets Ther. 10, 3867–3880. 10.2147/OTT.S133699 28814886 PMC5546812

[B68] Harrow S. Papanastassiou V. Harland J. Mabbs R. Petty R. Fraser M.. (2004). HSV1716 injection into the brain adjacent to tumour following surgical resection of high-grade glioma: safety data and long-term survival. Gene Ther. 11, 1648–1658. 10.1038/sj.gt.3302289 15334111

[B69] Hegi M. E. Diserens A. C. Gorlia T. Hamou M. F. de Tribolet N. Weller M.. (2005). MGMT gene silencing and benefit from temozolomide in glioblastoma. N. Engl. J. Med. 352, 997–1003. 10.1056/NEJMoa043331 15758010

[B70] Herrero Y. C. M. Cornelis J. J. Herold-Mende C. Rommelaere J. Schlehofer J. R. Geletneky K.. (2004). Parvovirus H-1 infection of human glioma cells leads to complete viral replication and efficient cell killing. Int. J. Cancer 109, 76–84. 10.1002/ijc.11626 14735471

[B71] Huang J. Zheng M. Zhang Z. Tang X. Chen Y. Peng A.. (2021). Interleukin-7-loaded oncolytic adenovirus improves CAR-T cell therapy for glioblastoma. Cancer Immunol. Immunother. 70, 2453–2465. 10.1007/s00262-021-02856-0 33543339 PMC10991970

[B72] Hygino da Cruz L. C. Jr. Rodriguez I. Domingues R. C. Gasparetto E. L. Sorensen A. G. (2011). Pseudoprogression and pseudoresponse: imaging challenges in the assessment of posttreatment glioma. Am. J. Neuroradiol. 32, 1978–1985. 10.3174/ajnr.A2397 21393407 PMC7964401

[B73] Ichikawa T. Petros W. P. Ludeman S. M. Fangmeier J. Hochberg F. H. Colvin O. M.. (2001). Intraneoplastic polymer-based delivery of cyclophosphamide for intratumoral bioconversion by a replicating oncolytic viral vector. Cancer Res. 61, 864–868. 11221871

[B74] Ito H. Aoki H. Kühnel F. Kondo Y. Kubicka S. Wirth T.. (2006). Autophagic cell death of malignant glioma cells induced by a conditionally replicating adenovirus. J. Natl. Cancer Inst. 98, 625–636. 10.1093/jnci/djj161 16670388

[B75] Jackson C. M. Lim M. Drake C. G. (2014). Immunotherapy for brain cancer: recent progress and future promise. Clin. Cancer Res. 20, 3651–3659. 10.1158/1078-0432.CCR-13-2057 24771646 PMC4729210

[B76] Jahan N. Lee J. M. Shah K. Wakimoto H. (2017). Therapeutic targeting of chemoresistant and recurrent glioblastoma stem cells with a proapoptotic variant of oncolytic herpes simplex virus. Int. J. Cancer 141, 1671–1681. 10.1002/ijc.30811 28567859 PMC5796532

[B77] Jain R. K. di Tomaso E. Duda D. G. Loeffler J. S. Sorensen A. G. Batchelor T. T.. (2007). Angiogenesis in brain tumours. Nat. Rev. Neurosci. 8, 610–622. 10.1038/nrn2175 17643088

[B78] Jiang H. Clise-Dwyer K. Ruisaard K. E. Fan X. Tian W. Gumin J.. (2014). Delta-24-RGD oncolytic adenovirus elicits anti-glioma immunity in an immunocompetent mouse model. PloS One 9:e97407. 10.1371/journal.pone.0097407 24827739 PMC4020829

[B79] Jiang H. Gomez-Manzano C. Aoki H. Alonso M. M. Kondo S. McCormick F.. (2007). Examination of the therapeutic potential of Delta-24-RGD in brain tumor stem cells: role of autophagic cell death. J. Natl. Cancer Inst. 99, 1410–1414. 10.1093/jnci/djm102 17848677

[B80] Jiang H. McCormick F. Lang F. F. Gomez-Manzano C. Fueyo J. (2006). Oncolytic adenoviruses as antiglioma agents. Expert Rev. Anticancer Ther. 6, 697–708. 10.1586/14737140.6.5.697 16759161

[B81] Jiang H. Rivera-Molina Y. Gomez-Manzano C. Clise-Dwyer K. Bover L. Vence L. M.. (2017). Oncolytic adenovirus and tumor-targeting immune modulatory therapy improve autologous cancer vaccination. Cancer Res. 77, 3894–3907. 10.1158/0008-5472.CAN-17-0468 28566332 PMC5549681

[B82] Josupeit R. Bender S. Kern S. Leuchs B. Hielscher T. Herold-Mende C.. (2016). Pediatric and adult high-grade glioma stem cell culture models are permissive to lytic infection with parvovirus H-1. Viruses 8:138. 10.3390/v8050138 27213425 PMC4885093

[B83] Kakiuchi Y. Kuroda S. Kanaya N. Kumon K. Tsumura T. Hashimoto M.. (2021). Local oncolytic adenovirotherapy produces an abscopal effect via tumor-derived extracellular vesicles. Mol. Ther. 29, 2920–2930. 10.1016/j.ymthe.2021.05.015 34023506 PMC8530926

[B84] Kanai R. Rabkin S. D. Yip S. Sgubin D. Zaupa C. M. Hirose Y.. (2012). Oncolytic virus-mediated manipulation of DNA damage responses: synergy with chemotherapy in killing glioblastoma stem cells. J. Natl. Cancer Inst. 104, 42–55. 10.1093/jnci/djr509 22173583 PMC3250384

[B85] Kanai R. Wakimoto H. Martuza R. L. Rabkin S. D. (2011). A novel oncolytic herpes simplex virus that synergizes with phosphoinositide 3-kinase/Akt pathway inhibitors to target glioblastoma stem cells. Clin. Cancer Res. 17, 3686–3696. 10.1158/1078-0432.CCR-10-3142 21505062 PMC3107877

[B86] Kang Y. A. Shin H. C. Yoo J. Y. Kim J. H. Kim J. S. Yun C. O.. (2008). Novel cancer antiangiotherapy using the VEGF promoter-targeted artificial zinc-finger protein and oncolytic adenovirus. Mol. Ther. 16, 1033–1040. 10.1038/mt.2008.63 18398429

[B87] Karachi A. Dastmalchi F. Mitchell D. A. Rahman M. (2018). Temozolomide for immunomodulation in the treatment of glioblastoma. Neuro Oncol. 20, 1566–1572. 10.1093/neuonc/noy072 29733389 PMC6231207

[B88] Kaufman H. L. Kohlhapp F. J. Zloza A. (2015). Oncolytic viruses: a new class of immunotherapy drugs. Nat. Rev. Drug Discov. 14, 642–662. 10.1038/nrd4663 26323545 PMC7097180

[B89] Kaufmann J. K. Nettelbeck D. M. (2012). Virus chimeras for Gene Ther., vaccination and oncolysis: adenoviruses and beyond. Trends Mol. Med. 18, 365–376. 10.1016/j.molmed.2012.04.008 22633438

[B500] Khoonkari M. Liang D. Kamperman M. Kruyt F. A. E. van Rijn P. (2022). Physics of brain cancer: Multiscale alterations of glioblastoma cells under extracellular matrix stiffening. Pharmaceutics 10:1031. 10.3390/pharmaceutics14051031 35631616 PMC9145282

[B90] Kieran M. W. Goumnerova L. Manley P. Chi S. N. Marcus K. J. Manzanera A. G.. (2019). Phase I study of gene-mediated cytotoxic immunotherapy with AdV-tk as adjuvant to surgery and radiation for pediatric malignant glioma and recurrent ependymoma. Neuro Oncol. 21, 537–546. 10.1093/neuonc/noy202 30883662 PMC6422437

[B91] Kiyokawa J. Kawamura Y. Ghouse S. M. Acar S. Barçın E. Martínez-Quintanilla J.. (2021). Modification of extracellular matrix enhances oncolytic adenovirus immunotherapy in glioblastoma. Clin. Cancer Res. 27, 889–902. 10.1158/1078-0432.CCR-20-2400 33257429 PMC7854507

[B93] Kleijn A. van den Bossche W. Haefner E. S. Belcaid Z. Burghoorn-Maas C. Kloezeman J. J.. (2017). The sequence of Delta24-RGD and TMZ administration in malignant glioma affects the role of CD8^+^T Cell anti-tumor activity. Mol. Ther. Oncolytics 5, 11–19. 10.1016/j.omto.2017.02.002 28480325 PMC5415315

[B94] Kmiecik J. Poli A. Brons N. H. Waha A. Eide G. E. Enger P.. (2013). Elevated CD3^+^ and CD8^+^ tumor-infiltrating immune cells correlate with prolonged survival in glioblastoma patients despite integrated immunosuppressive mechanisms in the tumor microenvironment and at the systemic level. J. Neuroimmunol. 264, 71–83. 10.1016/j.jneuroim.2013.08.013 24045166

[B95] Koks C. A. Garg A. D. Ehrhardt M. Riva M. Vandenberk L. Boon L.. (2015). Newcastle disease virotherapy induces long-term survival and tumor-specific immune memory in orthotopic glioma through the induction of immunogenic cell death. Int. J. Cancer 136, E313–E325. 10.1002/ijc.29202 25208916

[B96] Kunkel P. Ulbricht U. Bohlen P. Brockmann M. A. Fillbrandt R. Stavrou D.. (2001). Inhibition of glioma angiogenesis and growth *in vivo* by systemic treatment with a monoclonal antibody against vascular endothelial growth factor receptor-2. Cancer Res. 61, 6624–6628. 11559524

[B97] Kurozumi K. Hardcastle J. Thakur R. Yang M. Christoforidis G. Fulci G.. (2007). Effect of tumor microenvironment modulation on the efficacy of oncolytic virus therapy. J. Natl. Cancer Inst. 99, 1768–1781. 10.1093/jnci/djm229 18042934

[B98] Laborda E. Puig-Saus C. Cascalló M. Chillón M. Alemany R. (2013). Adeno-associated virus enhances wild-type and oncolytic adenovirus spread. Hum. Gene Ther. Methods 24, 372–380. 10.1089/hgtb.2013.124 24020980 PMC3869535

[B99] Lan Q. Xia S. Wang Q. Xu W. Huang H. Jiang S.. (2020). Development of oncolytic viro therapy: from genetic modification to combination therapy. Front. Med. 14, 160–184. 10.1007/s11684-020-0750-4 32146606 PMC7101593

[B100] Lang F. F. Conrad C. Gomez-Manzano C. Yung W. K. A. Sawaya R. Weinberg J. S.. (2018). Phase I study of DNX-2401 (Delta-24-RGD) oncolytic adenovirus: replication and immunotherapeutic effects in recurrent malignant glioma. J. Clin. Oncol. 36, 1419–1427. 10.1200/JCO.2017.75.8219 29432077 PMC6075856

[B101] Leber M. F. Bossow S. Leonard V. H. Zaoui K. Grossardt C. Frenzke M.. (2011). MicroRNA-sensitive oncolytic measles viruses for cancer-specific vector tropism. Mol. Ther. 19, 1097–1106. 10.1038/mt.2011.55 21468006 PMC3129790

[B102] Li B. Severson E. Pignon J. C. Zhao H. Li T. Novak J.. (2016). Comprehensive analyses of tumor immunity: implications for cancer immunotherapy. Genome Biol. 17:174. 10.1186/s13059-016-1028-7 27549193 PMC4993001

[B103] Li L. Liu S. Han D. Tang B. Ma J. (2020). Delivery and biosafety of oncolytic virotherapy. Front. Oncol. 10:475. 10.3389/fonc.2020.00475 32373515 PMC7176816

[B104] Li M. Li G. Kiyokawa J. Tirmizi Z. Richardson L. G. Ning J.. (2020). Characterization and oncolytic virus targeting of FAP-expressing tumor-associated pericytes in glioblastoma. Acta Neuropathol. Commun. 8:221. 10.1186/s40478-020-01096-0 33308315 PMC7730751

[B105] Li Y. Hofmann M. Wang Q. Teng L. Chlewicki L. K. Pircher H.. (2009). Structure of natural killer cell receptor KLRG1 bound to E-cadherin reveals basis for MHC-independent missing self recognition. Immunity 31, 35–46. 10.1016/j.immuni.2009.04.019 19604491 PMC3030123

[B106] Lichty B. D. Breitbach C. J. Stojdl D. F. Bell J. C. (2014). Going viral with cancer immunotherapy. Nat. Rev. Cancer 14, 559–567. 10.1038/nrc3770 24990523

[B107] Ligorio M. Sil S. Malagon-Lopez J. Nieman L. T. Misale S. Di Pilato M.. (2019). Stromal microenvironment shapes the intratumoral architecture of pancreatic cancer. Cell 178, 160–175.e127. 10.1016/j.cell.2019.05.012 31155233 PMC6697165

[B108] Lim M. Xia Y. Bettegowda C. Weller M. (2018). Current state of immunotherapy for glioblastoma. Nat. Rev. Clin. Oncol. 15, 422–442. 10.1038/s41571-018-0003-5 29643471

[B109] Lin L. T. Richardson C. D. (2016). The host cell receptors for measles virus and their interaction with the viral hemagglutinin (H) protein. Viruses 8:250. 10.3390/v8090250 27657109 PMC5035964

[B110] Logg C. R. Robbins J. M. Jolly D. J. Gruber H. E. Kasahara N. (2012). Retroviral replicating vectors in cancer. Methods Enzymol. 507, 199–228. 10.1016/B978-0-12-386509-0.00011-9 22365776 PMC7197216

[B111] Lohr J. Ratliff T. Huppertz A. Ge Y. Dictus C. Ahmadi R.. (2011). Effector T-cell infiltration positively impacts survival of glioblastoma patients and is impaired by tumor-derived TGF-β. Clin. Cancer Res. 17, 4296–4308. 10.1158/1078-0432.CCR-10-2557 21478334

[B112] Loskog A. (2015). Immunostimulatory gene therapy using oncolytic viruses as vehicles. Viruses 7, 5780–5791. 10.3390/v7112899 26561829 PMC4664972

[B113] Lucio-Eterovic A. K. Piao Y. de Groot J. F. (2009). Mediators of glioblastoma resistance and invasion during antivascular endothelial growth factor therapy. Clin. Cancer Res. 15, 4589–4599. 10.1158/1078-0432.CCR-09-0575 19567589

[B114] Ma R. Lu T. Li Z. Teng K. Y. Mansour A. G. Yu M.. (2021). An oncolytic virus expressing IL15/IL15Rα combined with off-the-shelf EGFR-CAR NK cells targets glioblastoma. Cancer Res. 81, 3635–3648. 10.1158/0008-5472.CAN-21-0035 34006525 PMC8562586

[B115] Mantovani A. Allavena P. (2015). The interaction of anticancer therapies with tumor-associated macrophages. J. Exp. Med. 212, 435–445. 10.1084/jem.20150295 25753580 PMC4387285

[B116] Markert J. M. Liechty P. G. Wang W. Gaston S. Braz E. Karrasch M.. (2009). Phase Ib trial of mutant herpes simplex virus G207 inoculated pre-and post-tumor resection for recurrent GBM. Mol. Ther. 17, 199–207. 10.1038/mt.2008.228 18957964 PMC2834981

[B117] Martikainen M. Essand M. (2019). Virus-based immunotherapy of glioblastoma. Cancers 11, 16–186. 10.3390/cancers11020186 30764570 PMC6407011

[B118] Mazzacurati L. Marzulli M. Reinhart B. Miyagawa Y. Uchida H. Goins W. F.. (2015). Use of miRNA response sequences to block off-target replication and increase the safety of an unattenuated, glioblastoma-targeted oncolytic HSV. Mol. Ther. 23, 99–107. 10.1038/mt.2014.177 25200130 PMC4426800

[B119] Meier A. F. Fraefel C. Seyffert M. (2020). The interplay between adeno-associated virus and its helper viruses. Viruses 12:662. 10.3390/v12060662 32575422 PMC7354565

[B120] Merrill M. K. Bernhardt G. Sampson J. H. Wikstrand C. J. Bigner D. D. Gromeier M.. (2004). Poliovirus receptor CD155-targeted oncolysis of glioma. Neuro Oncol. 6, 208–217. 10.1215/S1152851703000577 15279713 PMC1871993

[B121] Mezhir J. J. Advani S. J. Smith K. D. Darga T. E. Poon A. P. Schmidt H.. (2005). Ionizing radiation activates late herpes simplex virus 1 promoters via the p38 pathway in tumors treated with oncolytic viruses. Cancer Res. 65, 9479–9484. 10.1158/0008-5472.CAN-05-1927 16230412

[B122] Mineta T. Rabkin S. D. Yazaki T. Hunter W. D. Martuza R. L. (1995). Attenuated multi-mutated herpes simplex virus-1 for the treatment of malignant gliomas. Nat. Med. 1, 938–943. 10.1038/nm0995-938 7585221

[B123] Mitchell L. A. Lopez Espinoza F. Mendoza D. Kato Y. Inagaki A. Hiraoka K.. (2017). Toca 511 gene transfer and treatment with the prodrug, 5-fluorocytosine, promotes durable antitumor immunity in a mouse glioma model. Neuro Oncol. 19, 930–939. 10.1093/neuonc/nox037 28387849 PMC5570153

[B124] Miyauchi J. T. Chen D. Choi M. Nissen J. C. Shroyer K. R. Djordevic S.. (2016). Ablation of neuropilin 1 from glioma-associated microglia and macrophages slows tumor progression. Oncotarget 7, 9801–9814. 10.18632/oncotarget.6877 26755653 PMC4891085

[B125] Moon C. Y. Choi J. W. Kasala D. Jung S. J. Kim S. W. Yun C. O.. (2015). Dual tumor targeting with pH-sensitive and bioreducible polymer-complexed oncolytic adenovirus. Biomaterials 41, 53–68. 10.1016/j.biomaterials.2014.11.021 25522965

[B126] Mormino A. Garofalo S. (2022). Dialogue among lymphocytes and microglia in glioblastoma microenvironment. Cancers (Basel) 14:2632. 10.3390/cancers14112632 35681612 PMC9179556

[B127] Nair S. Mazzoccoli L. Jash A. Govero J. Bais S. S. Hu T.. (2020). Zika virus oncolytic activity requires CD8^+^ T cells and is boosted by immune checkpoint blockade. JCI Insight 6:e144619. 10.1172/jci.insight.144619 33232299 PMC7821591

[B128] Nakashima H. Kaufmann J. K. Wang P. Y. Nguyen T. Speranza M. C. Kasai K.. (2015). Histone deacetylase 6 inhibition enhances oncolytic viral replication in glioma. J. Clin. Invest. 125, 4269–4280. 10.1172/JCI80713 26524593 PMC4639993

[B129] Nicolas A. Jolinon N. Alazard-Dany N. Barateau V. Epstein A. L. Greco A.. (2012). Factors influencing helper-independent adeno-associated virus replication. Virology 432, 1–9. 10.1016/j.virol.2012.05.027 22727829

[B130] Nishikawa R. Ji X. D. Harmon R. C. Lazar C. S. Gill G. N. Cavenee W. K.. (1994). A mutant epidermal growth factor receptor common in human glioma confers enhanced tumorigenicity. Proc. Natl. Acad. Sci. U S A 91, 7727–7731. 10.1073/pnas.91.16.7727 8052651 PMC44475

[B131] Nishio N. Dotti G. (2015). Oncolytic virus expressing RANTES and IL-15 enhances function of CAR-modified T cells in solid tumors. OncoImmunology 4:e988098. 10.4161/21505594.2014.988098 25949885 PMC4404887

[B132] Omuro A. Vlahovic G. Lim M. Sahebjam S. Baehring J. Cloughesy T.. (2018). Nivolumab with or without ipilimumab in patients with recurrent glioblastoma: results from exploratory phase I cohorts of CheckMate 143. Neuro Oncol. 20, 674–686. 10.1093/neuonc/nox208 29106665 PMC5892140

[B134] O’Rourke D. M. Nasrallah M. P. Desai A. Melenhorst J. J. Mansfield K. Morrissette J. J. D.. (2017). A single dose of peripherally infused EGFRvIII-directed CAR T cells mediates antigen loss and induces adaptive resistance in patients with recurrent glioblastoma. Sci. Transl. Med. 9:eaaa0984. 10.1126/scitranslmed.aaa0984 28724573 PMC5762203

[B135] Otvos B. Silver D. J. Mulkearns-Hubert E. E. Alvarado A. G. Turaga S. M. Sorensen M. D.. (2016). Cancer stem cell-secreted macrophage migration inhibitory factor stimulates myeloid derived suppressor cell function and facilitates glioblastoma immune evasion. Stem Cells (Dayton, Ohio) 34, 2026–2039. 10.1002/stem.2393 27145382 PMC5820763

[B136] Papa M. P. Meuren L. M. Coelho S. V. A. Lucas C. G. O. Mustafá Y. M. Lemos Matassoli F.. (2017). Zika virus infects, activates and crosses brain microvascular endothelial cells, without barrier disruption. Front. Microbiol. 8:2557. 10.3389/fmicb.2017.02557 29312238 PMC5743735

[B137] Patel D. M. Foreman P. M. Nabors L. B. Riley K. O. Gillespie G. Y. Markert J. M.. (2016). Design of a phase I clinical trial to evaluate M032, a genetically engineered HSV-1 expressing IL-12, in patients with recurrent/progressive glioblastoma multiforme, anaplastic astrocytoma, or gliosarcoma. Hum. Gene Ther. Clin. Dev. 27, 69–78. 10.1089/humc.2016.031 27314913 PMC4932657

[B138] Peereboom D. M. Alban T. J. Grabowski M. M. Alvarado A. G. Otvos B. Bayik D.. (2019). Metronomic capecitabine as an immune modulator in glioblastoma patients reduces myeloid-derived suppressor cells. JCI Insight 4:e130748. 10.1172/jci.insight.130748 31600167 PMC6948860

[B139] Peng W. Chen J. Q. Liu C. Malu S. Creasy C. Tetzlaff M. T.. (2016). Loss of PTEN promotes resistance to T Cell-mediated immunotherapy. Cancer Discov. 6, 202–216. 10.1158/2159-8290.CD-15-0283 26645196 PMC4744499

[B140] Perez O. D. Logg C. R. Hiraoka K. Diago O. Burnett R. Inagaki A.. (2012). Design and selection of Toca 511 for clinical use: modified retroviral replicating vector with improved stability and gene expression. Mol. Ther. 20, 1689–1698. 10.1038/mt.2012.83 22547150 PMC3437576

[B141] Peters C. Paget M. Tshilenge K. T. Saha D. Antoszczyk S. Baars A.. (2018). Restriction of replication of oncolytic herpes simplex virus with a deletion of γ34.5 in glioblastoma stem-like cells. J. Virol. 92, e00246–e00218. 10.1128/JVI.00246-18 29793956 PMC6052301

[B142] Petralia F. Tignor N. Reva B. Koptyra M. Chowdhury S. Rykunov D.. (2020). Integrated proteogenomic characterization across major histological types of pediatric brain cancer. Cell 183, 1962–1985.e31. 10.1016/j.cell.2020.10.044 33242424 PMC8143193

[B143] Preusser M. Lim M. Hafler D. A. Reardon D. A. Sampson J. H. (2015). Prospects of immune checkpoint modulators in the treatment of glioblastoma. Nat. Rev. Neurol. 11, 504–514. 10.1038/nrneurol.2015.139 26260659 PMC4782584

[B144] Qiao J. Dey M. Chang A. L. Kim J. W. Miska J. Ling A.. (2015). Intratumoral oncolytic adenoviral treatment modulates the glioma microenvironment and facilitates systemic tumor-antigen-specific T cell therapy. Oncoimmunology 4:e1022302. 10.1080/2162402X.2015.1022302 26405578 PMC4570114

[B145] Qiao J. Wang H. Kottke T. White C. Twigger K. Diaz R. M.. (2008). Cyclophosphamide facilitates antitumor efficacy against subcutaneous tumors following intravenous delivery of reovirus. Clin. Cancer Res. 14, 259–269. 10.1158/1078-0432.CCR-07-1510 18172278 PMC3046731

[B146] Quail D. F. Joyce J. A. (2017). The microenvironmental landscape of brain tumors. Cancer Cell 31, 326–341. 10.1016/j.ccell.2017.02.009 28292436 PMC5424263

[B147] Rampling R. Peoples S. Mulholland P. J. James A. Al-Salihi O. Twelves C. J.. (2016). A cancer research UK first time in human phase I trial of IMA950 (Novel Multipeptide Therapeutic Vaccine) in patients with newly diagnosed glioblastoma. Clin. Cancer Res. 22, 4776–4785. 10.1158/1078-0432.CCR-16-0506 27225692 PMC5026298

[B148] Reardon D. A. Gokhale P. C. Klein S. R. Ligon K. L. Rodig S. J. Ramkissoon S. H.. (2016). Glioblastoma eradication following immune checkpoint blockade in an orthotopic, immunocompetent model. Cancer Immunol. Res. 4, 124–135. 10.1158/2326-6066.CIR-15-0151 26546453

[B149] Romero D. (2021). HSV-1 G207 is active in paediatric glioma. Nat. Rev. Clin. oncol. 18:321. 10.1038/s41571-021-00515-y 33911216

[B150] Saha D. Martuza R. L. Rabkin S. D. (2017). Macrophage polarization contributes to glioblastoma eradication by combination immunovirotherapy and immune checkpoint blockade. Cancer Cell 32, 253–267.e5. 10.1016/j.ccell.2017.07.006 28810147 PMC5568814

[B151] Saha D. Wakimoto H. Peters C. W. Antoszczyk S. J. Rabkin S. D. Martuza R. L.. (2018). Combinatorial effects of VEGFR kinase inhibitor axitinib and oncolytic virotherapy in mouse and human glioblastoma stem-like cell models. Clin. Cancer Res. 24, 3409–3422. 10.1158/1078-0432.CCR-17-1717 29599413 PMC6050085

[B152] Samson A. Scott K. J. Taggart D. West E. J. Wilson E. Nuovo G. J.. (2018). Intravenous delivery of oncolytic reovirus to brain tumor patients immunologically primes for subsequent checkpoint blockade. Sci. Transl. Med. 10:eaam7577. 10.1126/scitranslmed.aam7577 29298869 PMC6276984

[B153] Sette P. Amankulor N. Li A. Marzulli M. Leronni D. Zhang M.. (2019). GBM-targeted oHSV armed with matrix metalloproteinase 9 enhances anti-tumor activity and animal survival. Mol. Ther. Oncolytics 15, 214–222. 10.1016/j.omto.2019.10.005 31890868 PMC6926261

[B154] Shah A. C. Parker J. N. Gillespie G. Y. Lakeman F. D. Meleth S. Markert J. M.. (2007). Enhanced antiglioma activity of chimeric HCMV/HSV-1 oncolytic viruses. Gene Ther. 14, 1045–1054. 10.1038/sj.gt.3302942 17429445

[B155] Shinoura N. Yoshida Y. Tsunoda R. Ohashi M. Zhang W. Asai A.. (1999). Highly augmented cytopathic effect of a fiber-mutant E1B-defective adenovirus for gene therapy of gliomas. Cancer Res. 59, 3411–3416. 10416603

[B156] Sinkovics J. G. Horvath J. C. (2000). Newcastle disease virus (NDV): brief history of its oncolytic strains. J. Clin. Virol. 16, 1–15. 10.1016/s1386-6532(99)00072-4 10680736

[B157] Stupp R. Mason W. P. van den Bent M. J. Weller M. Fisher B. Taphoorn M. J.. (2005). Radiotherapy plus concomitant and adjuvant temozolomide for glioblastoma. N. Engl. J. Med. 352, 987–996. 10.1056/NEJMoa043330 15758009

[B158] Su K. Y. Balasubramaniam V. R. M. T. (2019). Zika virus as oncolytic therapy for brain cancer: myth or reality? Front. Microbiol. 10:2715. 10.3389/fmicb.2019.02715 31824472 PMC6879458

[B159] Suh C. H. Kim H. S. Jung S. C. Park J. E. Choi C. G. Kim S. J.. (2019). MRI as a diagnostic biomarker for differentiating primary central nervous system lymphoma from glioblastoma: a systematic review and meta-analysis. J. Magn. Reson. Imaging 50, 560–572. 10.1002/jmri.26602 30637843

[B162] Tahara H. Lotze M. T. (1995). Antitumor effects of interleukin-12 (IL-12): applications for the immunotherapy and gene therapy of cancer. Gene Ther. 2, 96–106. 7719935

[B160] Tähtinen S. Blattner C. Vähä-Koskela M. Saha D. Siurala M. Parviainen S.. (2016). T-cell therapy enabling adenoviruses coding for IL2 and TNFα induce systemic immunomodulation in mice with spontaneous melanoma. J. Immunother. 39, 343–354. 10.1097/CJI.0000000000000144 27741089

[B161] Tähtinen S. Grönberg-Vähä-Koskela S. Lumen D. Merisalo-Soikkeli M. Siurala M. Airaksinen A. J.. (2015). Adenovirus improves the efficacy of adoptive T-cell therapy by recruiting immune cells to and promoting their activity at the tumor. Cancer Immunol. Res. 3, 915–925. 10.1158/2326-6066.CIR-14-0220-T 25977260

[B163] Thaci B. Ulasov I. V. Ahmed A. U. Ferguson S. D. Han Y. Lesniak M. S.. (2013). Anti-angiogenic therapy increases intratumoral adenovirus distribution by inducing collagen degradation. Gene Ther. 20, 318–327. 10.1038/gt.2012.42 22673390 PMC3443547

[B164] Tian Y. Xie D. Yang L. (2022). Engineering strategies to enhance oncolytic viruses in cancer immunotherapy. Signal Transduct. Target. Ther. 7:117. 10.1038/s41392-022-00951-x 35387984 PMC8987060

[B165] Tomita Y. Kurozumi K. Yoo J. Y. Fujii K. Ichikawa T. Matsumoto Y.. (2019). Oncolytic herpes virus armed with vasculostatin in combination with bevacizumab abrogates glioma invasion via the CCN1 and AKT signaling pathways. Mol. Cancer Ther. 18, 1418–1429. 10.1158/1535-7163.MCT-18-0799 31092561

[B166] Topalian S. L. Taube J. M. Anders R. A. Pardoll D. M. (2016). Mechanism-driven biomarkers to guide immune checkpoint blockade in cancer therapy. Nat. Rev. Cancer 16, 275–287. 10.1038/nrc.2016.36 27079802 PMC5381938

[B167] Ulasov I. V. Zhu Z. B. Tyler M. A. Han Y. Rivera A. A. Khramtsov A.. (2007). Survivin-driven and fiber-modified oncolytic adenovirus exhibits potent antitumor activity in established intracranial glioma. Hum. Gene Ther. 18, 589–602. 10.1089/hum.2007.002 17630837

[B168] van den Bossche W. B. L. Kleijn A. Teunissen C. E. Voerman J. S. A. Teodosio C. Noske D. P.. (2018). Oncolytic Virother.apy in glioblastoma patients induces a tumor macrophage phenotypic shift leading to an altered glioblastoma microenvironment. Neuro Oncol. 20, 1494–1504. 10.1093/neuonc/noy082 29796615 PMC6176807

[B170] Vasileva N. Ageenko A. Dmitrieva M. Nushtaeva A. Mishinov S. Kochneva G.. (2021). Double recombinant vaccinia virus: a candidate drug against human glioblastoma. Life (Basel) 11:1084. 10.3390/life11101084 34685455 PMC8538059

[B171] Verhaak R. G. W. Hoadley K. A. Purdom E. Wang V. Qi Y. Wilkerson M. D.. (2010). Integrated genomic analysis identifies clinically relevant subtypes of glioblastoma characterized by abnormalities in PDGFRA, IDH1, EGFR and NF1. Cancer Cell 17, 98–110. 10.1016/j.ccr.2009.12.020 20129251 PMC2818769

[B172] Webster R. J. Giles K. M. Price K. J. Zhang P. M. Mattick J. S. Leedman P. J. (2009). Regulation of epidermal growth factor receptor signaling in human cancer cells by microRNA-7. J. Biol. Chem. 284, 5731–5741. 10.1074/jbc.M804280200 19073608

[B173] Weiss T. Puca E. Silginer M. Hemmerle T. Pazahr S. Bink A.. (2020). Immunocytokines are a promising immunotherapeutic approach against glioblastoma. Sci. Transl. Med. 12:eabb2311. 10.1126/scitranslmed.abb2311 33028706

[B174] Whatcott C. J. Han H. Posner R. G. Hostetter G. Von Hoff D. D. (2011). Targeting the tumor microenvironment in cancer: why hyaluronidase deserves a second look. Cancer Discov. 1, 291–296. 10.1158/2159-8290.CD-11-0136 22053288 PMC3204883

[B175] Wilcox M. E. Yang W. Senger D. Rewcastle N. B. Morris D. G. Brasher P. M.. (2001). Reovirus as an oncolytic agent against experimental human malignant gliomas. J. Natl. Cancer Inst. 93, 903–912. 10.1093/jnci/93.12.903 11416111

[B176] Wojton J. Kaur B. (2010). Impact of tumor microenvironment on oncolytic viral therapy. Cytokine Growth Factor Rev. 21, 127–134. 10.1016/j.cytogfr.2010.02.014 20399700 PMC2881175

[B177] Wollmann G. Ozduman K. van den Pol A. N. (2012). Oncolytic virus therapy for glioblastoma multiforme: concepts and candidates. Cancer J. 18, 69–81. 10.1097/PPO.0b013e31824671c9 22290260 PMC3632333

[B178] Wortmann A. Vöhringer S. Engler T. Corjon S. Schirmbeck R. Reimann J.. (2008). Fully detargeted polyethylene glycol-coated adenovirus vectors are potent genetic vaccines and escape from pre-existing anti-adenovirus antibodies. Mol. Ther. 16, 154–162. 10.1038/sj.mt.6300306 17848961

[B179] Xing F. Xiao J. Wu J. Liang J. Lu X. Guo L.. (2021). Modulating the tumor microenvironment via oncolytic virus and PI3K inhibition synergistically restores immune checkpoint therapy response in PTEN-deficient glioblastoma. Signal Transduct. Target. Ther. 6:275. 10.1038/s41392-021-00609-0 34315854 PMC8316409

[B181] Xu X. Chen W. Zhu W. Chen J. Ma B. Ding J.. (2021). Adeno-associated virus (AAV)-based gene therapy for glioblastoma. Cancer Cell Int. 21:76. 10.1186/s12935-021-01776-4 33499886 PMC7836184

[B180] Xu B. Ma R. Russell L. Yoo J. Y. Han J. Cui H.. (2018). An oncolytic herpesvirus expressing E-cadherin improves survival in mouse models of glioblastoma. Nat. Biotechnol. 10.1038/nbt.4302. [Online ahead of print]. 30475349 PMC6535376

[B182] Yanagi Y. (2001). The cellular receptor for measles virus–elusive no more. Rev. Med. Virol. 11, 149–156. 10.1002/rmv.308 11376478

[B183] Ye L. Park J. J. Dong M. B. Yang Q. Chow R. D. Peng L.. (2019). *in vivo* CRISPR screening in CD8 T cells with AAV-sleeping beauty hybrid vectors identifies membrane targets for improving immunotherapy for glioblastoma. Nat. Biotechnol. 37, 1302–1313. 10.1038/s41587-019-0246-4 31548728 PMC6834896

[B184] Yin J. Kim S. S. Choi E. Oh Y. T. Lin W. Kim T. H.. (2020). ARS2/MAGL signaling in glioblastoma stem cells promotes self-renewal and M2-like polarization of tumor-associated macrophages. Nat. Commun. 11:2978. 10.1038/s41467-020-16789-2 32532977 PMC7293269

[B185] Yong R. L. Shinojima N. Fueyo J. Gumin J. Vecil G. G. Marini F. C.. (2009). Human bone marrow-derived mesenchymal stem cells for intravascular delivery of oncolytic adenovirus Δ24-RGD to human gliomas. Cancer Res. 69, 8932–8940. 10.1158/0008-5472.CAN-08-3873 19920199 PMC2789204

[B186] Yoo J. Y. Swanner J. Otani Y. Nair M. Park F. Banasavadi-Siddegowda Y.. (2019). Oncolytic HSV therapy increases trametinib access to brain tumors and sensitizes them *in vivo*. Neuro Oncol. 21, 1131–1140. 10.1093/neuonc/noz079 31063549 PMC7571492

[B188] Zhang W. Fulci G. Buhrman J. S. Stemmer-Rachamimov A. O. Chen J. W. Wojtkiewicz G. R.. (2012). Bevacizumab with angiostatin-armed oHSV increases antiangiogenesis and decreases bevacizumab-induced invasion in U87 glioma. Mol. Ther. 20, 37–45. 10.1038/mt.2011.187 21915104 PMC3255598

[B189] Zhang W. Fulci G. Wakimoto H. Cheema T. A. Buhrman J. S. Jeyaretna D. S.. (2013). Combination of oncolytic herpes simplex viruses armed with angiostatin and IL-12 enhances antitumor efficacy in human glioblastoma models. Neoplasia 15, 591–599. 10.1593/neo.13158 23730207 PMC3664991

[B187] Zhang M. Hutter G. Kahn S. A. Azad T. D. Gholamin S. Xu C. Y.. (2016). Anti-CD47 treatment stimulates phagocytosis of glioblastoma by M1 and M2 polarized macrophages and promotes M1 polarized macrophages *in vivo*. PLoS One 11:e0153550. 10.1371/journal.pone.0153550 27092773 PMC4836698

[B190] Zhao W. J. Fan Y. P. Ou G. Y. Qiao X. Y. (2022). LASS2 impairs proliferation of glioma stem cells and migration and invasion of glioma cells mainly via inhibition of EMT and apoptosis promotion. J. Cancer 13, 2281–2292. 10.7150/jca.71256 35517425 PMC9066216

[B191] Zhou F. (2009). Molecular mechanisms of IFN-γ to up-regulate MHC class I antigen processing and presentation. Int. Rev. Immunol. 28, 239–260. 10.1080/08830180902978120 19811323

[B192] Zhou Y. Xu L. Wang Z. Liu H. Zhang X. Shu C.. (2022). Sequentially targeting and intervening mutual Polo-like Kinase 1 on CAFs and tumor cells by dual targeting nano-platform for cholangiocarcinoma treatment. Theranostics 12, 3911–3927. 10.7150/thno.70557 35664077 PMC9131280

[B193] Zhu Z. Gorman M. J. McKenzie L. D. Chai J. N. Hubert C. G. Prager B. C.. (2017). Zika virus has oncolytic activity against glioblastoma stem cells. J. Exp. Med. 214, 2843–2857. 10.1084/jem.20171093 28874392 PMC5626408

[B194] Zhu Z. Mesci P. Bernatchez J. A. Gimple R. C. Wang X. Schafer S. T.. (2020). Zika virus targets glioblastoma stem cells through a SOX2-integrin α(v)β(5) axis. Cell Stem Cell 26, 187–204.e10. 10.1016/j.stem.2019.11.016 31956038 PMC9628766

